# Supplementary material for: Developmental factors associated with decline in grip strength from midlife to old age: a British birth cohort study
Source: BMJ Open. 2019 May 9;9(5):e025755. doi: 10.1136/bmjopen-2018-025755 (PMC6528009; doi:10.1136/bmjopen-2018-025755)
Supplement: Supplementary file 1 [file bmjopen-2018-025755supp001.pdf]

Developmental factors associated with decline in grip strength from midlife to old age: a British birth cohort study

Kuh D[1], Hardy R, Blodgett JM[1], Cooper R[1]

[1]MRC Unit for Lifelong Health and Ageing at UCL, London

SUPPLEMENTARY MATERIAL

Supplementary Material

Model specification

Multilevel models were used to estimate the grip strength trajectory defined by the intercept (at age 53) and the slope, with measurements (level1) nested within individuals (level 2).

If $grip_{ij}$ denotes grip strength of individual *j (j=1,…,n)* on measurement occasion *i (i=1,2,3)* and age_{ij} the age (centred at 53 years) at which that measurement was recorded, the basic multilevel model can be written as:

$$grip_{ij} = \alpha + u_j + (\beta_1 + v_j)age_{ij} + e_{ij}$$

where the fixed parameter α is the mean intercept and the fixed parameter β the mean slope. The parameters u_j and v_j represent the deviation of the jth individual's intercept from the overall mean and slope, respectively. Finally, e_{ij} is the residual error term.

Covariates were then added to the model and could be associated with the intercept and the slope. Covariate by age interactions were assessed and included in models if p<0.1.

For Table 3 in men, the first line of the table is from the model:

$$\begin{aligned} grip_{ij} &= \alpha + u_j + (\beta_1 + v_j)age_{ij} + \beta_2 bwt_j \\ &+ \beta_3 htsize_j + \beta_4 httempo_j + \beta_5 htvelocity_j + \beta_6 wtsize_j \\ &+ \beta_7 wttempo_j + \beta_8 wtvelocity_j + \beta_9 adultht_{ij} + e_{ij} \end{aligned}$$

Adjusting for BMI, for example, as a time-varying covariate which is associated with both intercept (β_{10}, β_{11}) and slope (γ_1), the model is:

$$\begin{aligned} grip_{ij} &= \alpha + u_j + (\beta_1 + v_j)age_{ij} + \beta_2 bwt_j \\ &+ \beta_3 htsize_j + \beta_4 httempo_j + \beta_5 htvelocity_j + \beta_6 wtsize_j \\ &+ \beta_7 wttempo_j + \beta_8 wtvelocity_j + \beta_9 adultht_{ij} + \beta_{10} BMI_{ij} + \beta_{11} BMI_{ij}^2 \\ &+ \gamma_1 BMI_{ij}.age_{ij} + e_{ij} \end{aligned}$$

Similarly, for Table 4 in men, the first line of the table where childhood cognition is associated with both intercept (β_2, β_3) and the slope (γ_1), the model is:

$$\begin{aligned} grip_{ij} &= \alpha + u_j + (\beta_1 + v_j)age_{ij} + \beta_2 childcog_j + \beta_3 childcog_j^2 \\ &+ \gamma_1 childcog_j.age_{ij} + \beta_4 walking_j + \beta_5 walking_j^2 + \gamma_2 walking_j.age_{ij} \\ &+ \beta_6 adultht_{ij} + e_{ij} \end{aligned}$$

Adjusting for adult BMI:

$$grip_{ij} = \alpha + u_j + (\beta_1 + v_j)age_{ij} + \beta_2 childcog_j + \beta_3 childcog_j^2 + \gamma_1 childcog_j.age_{ij} + \beta_4 walking_j + \beta_5 walking_j^2 + \gamma_2 walking_j.age_{ij} + \beta_6 adultht_{ij} + \beta_7 BMI_{ij} + \beta_8 BMI_{ij}^2 + \gamma_3 BMI_{ij}.age_{ij} + e_{ij}$$

For Table 5 in women, the first line is from the model:

$$grip_{ij} = \alpha + u_j + (\beta_1 + v_j)age_{ij} + \beta_2 bwt_j + \gamma_1 bwt_j.age_{ij} + \beta_3 childcog_j + \beta_4 walking_j + \beta_5 adultht_{ij} + e_{ij}$$

Adjusting for adult BMI:

$$grip_{ij} = \alpha + u_j + (\beta_1 + v_j)age_{ij} + \beta_2 bwt_j + \gamma_1 bwt_j.age_{ij} + \beta_3 childcog_j + \beta_4 walking_j + \beta_5 adultht_{ij} + \beta_6 BMI_{ij} + \beta_7 BMI_{ij}^2 + \gamma_2 BMI_{ij}.age_{ij} + e_{ij}$$

Supplementary Table 1. Estimates From Multilevel Models Showing Mean Differences in Grip Strength (kg) in 1,375 NSHD Men (3,111 Observations) and in 1,385 NSHD Women (3,266 Observations) for Mutually Adjusted Physical Growth Indicators. All Models Adjusted for Age Term and Adult height and Using Sample With Complete Childhood Data.

	MEN			WOMEN		
	Reg. Coeff.	95% CI	P-value	Reg. Coeff.	95% CI	P-value
Age (months) at sitting	0.030	-0.35,0.40	0.9	-0.031	-0.27,0.21	0.8
Age (months) at standing	0.14	-0.23,0.46	0.6	-0.29	-0.53,-0.052	0.02
Age (months) at walking						
Walking	1.72	0.33,3.1	0.01	0.11	-0.093,0.32	0.3
$Walking^2$	-0.075	-0.12,-0.028	0.002	N/A		

Supplementary Table 2. Estimates From Multilevel Models Showing Mean Differences in Grip Strength (kg) and Mean Differences in Grip Strength Change (kg/year) in 1,316 NSHD Men (2,983 Observations) for Mutually Adjusted Childhood Factors. All Models Adjusted for Age Term and Standardised Adult Height.

	Adjusted for age term and height			Fully adjusted		
	Reg. Coeff.	95% CI	P-value+	Reg. Coeff.	95% CI	P-value+
BIRTHWEIGHT (kg)	2.00	1.05,2.95	<0.001	1.40	0.41,2.38	0.005
GROWTH PARAMETERS						
Height -size (cm)	-0.09	-0.32,0.14	0.4	-0.07	-0.30,0.16	0.5
Height-tempo (%)	0.16	0.052,0,26	0.004	0.15	0.046,0.26	0.005
Height-velocity (%)	-0.02	-0.10,0.06	0.6	-0.03	-0.10,0.05	0.5
Weight-size (kg)	0.93	0.41,1.44	<0.001	0.62	0.094,1.15	0.02
Weight-tempo (%)	-0.07	-0.13,0.00	0.051	-0.05	-0.12,0.02	0.1
Weight-velocity (%)	-0.10	-0.18,-0.02	0.011	-6.16	-14.11,1.79	0.1
MOTOR DEVELOPMENT (months)						
Age at walking						
Walking	1.35	-0.073,2.78	0.06	1.30	-0.11,2.72	0.07
Walking^2	-0.057	-0.10,-0.01	0.02	-0.054	-0.10,-0.01	0.02
CHILDHOOD DEVELOPMENT (SD)						
Cognition	-0.32	-0.98,0.33	0.3	-0.41	-1.08,0.27	0.2
Cognition^2	-0.78	-1.25,-0.30	0.001	-0.77	-1.24,-0.30	0.001
Cognition*age (year)	0.07	0.021,0.11	0.004	0.067	0.02,0.11	0.003
Cognition^2 * age (year)	0.03	-0.01,0.06	0.09	0.024	-0.01,0.06	0.2
FATHER'S OCCUPATIONAL CLASS						
I & II	REF		0.08	REF		0.09
III	-0.62	-1.81,0.58		-0.77	-1.99,0.44	
IV and V	-1.55	-2.91,-0.18		-1.63	-3.08,-0.18	

+ P-values are given for the overall tests of associations

Supplementary Table 3. Estimates From Multilevel Models Showing Mean Differences in Grip Strength (kg) and Mean Differences in Grip Strength Change (kg/year) in 1,320 NSHD Women (3,069 observations) for Mutually Adjusted Childhood Factors. All Models Adjusted for Age Term and Standardised Adult Height.

	Adjusted for age term and height			Adjusted for age term and height and all childhood factors		
	Reg. Coeff.	95% CI	P-value+	Reg. Coeff.	95% CI	P-value+
BIRTHWEIGHT (kg)						
Birthweight	1.20	0.32,2.08	.007	1.05	0.24,1.86	0.01
Birthweight*age (year)	-0.07	-0.13,-0.002	.04	-0.07	-0.14,-0.01	0.03
GROWTH PARAMETERS						
Height -size (cm)	-0.14	-0.28,0.01	0.07	-0.15	-0.30,0.01	0.06
Height-tempo (%)	0.02	-0.041,0.08	0.6	0.02	-0.04,0.09	0.3
Height-velocity (%)	-0.01	-0.05,0.037	0.7	-0.01	-0.05,0.04	0.9
Weight-size (kg)	0.36	-0.02,0.75	0.06	0.25	-0.15,0.65	0.2
Weight-tempo (%)	0.00	-0.05,0.05	1.0	0.01	-0.05,0.06	0.8
Weight-velocity (%)	-0.05	-0.11,0.07	0.08	-0.03	-0.91,0.26	0.3
MOTOR DEVELOPMENT (months)						
Age at standing	-0.16	2.50,3.39	0.03	-0.14	-0.29,0.00	0.06
CHILDHOOD DEVELOPMENT (SD)						
Cognition	0.41	0.09,0.73	.01	0.42	0.07,0.78	0.02
FATHER’S OCCUPATIONAL CLASS						
I & II	REF		0.2	REF		0.2
III	-0.59	-1.65,0.47		-0.41	-1.38,0.57	
IV and V	0.22	-0.99,1.43		0.59	-0.55,1.72	
III *age (year)	-0.02	-0.10,0.06	0.2	-0.02	-0.10,0.05	0.1
IV and V*age (year)	-0.08	-0.17,0.01		-0.09	-0.18,0.004	

+ P-values are given for the overall tests of associations

Supplementary Table 4 Estimates From Multilevel Models Showing Mean Differences in Grip Strength (kg) and Mean Differences in Grip Strength Change (kg/year) by Each Adult Factor. All Models Adjusted for Age Term and Standardised Adult Height.

		Men					Women	
	n	Reg. Coeff.	95% CI	P-value	n	Reg. Coeff.	95% CI	P-value
a.BMI (per SD)[a]	1,528				1,524			
BMI		1.66	0.93,2.38	<0.001		-0.18	-0.42,0.049	0.1
BMI^2		-0.49	-0.80,-0.18	0.002		n/a		
BMI*age		-0.057	-0.11,-0.0056	0.03		n/a		
b. Health conditions at 53y	1,415				1,441			
Health conditions		-0.84	-1.70,0.19	0.05		-1.42	-1.89,-0.94	<0.001
Health $conditions^2$		-0.055	-0.12,0.0057	0.08		0.030	-0.0066,0.066	0.1
c. Smoker v. non-smoker [a]	1,516	-1.28	-2.38,-0.18	0.02	1,520	-0.17	-0.90,0.55	0.6
d. Leisure time physical activity[a]	1,521					1522		
Inactive		REF		0.003		REF		0.005
Intermediate		1.52	0.54,2.51			0.85	0.21,1.48	
Active		1.04	0.23,1.84			0.71	0.18,1.24	
e. Verbal memory[a]	1,489				1,508			
Verbal memory		-0.67	-1.24,-0.093	0.02		0.070	-0.31,0.45	0.7
Verbal memory*age		0.10	0.061,0.15	<0.001		0.032	0.0020,0.063	0.04
f. Qualifications (per step)	1,445	0.17	-0.13,0.48	0.3	1,444	0.38	0.16,0.59	0.001
g. Own social class at 53y[b]	1,511				1,518			
I and II		REF		0.001		REF		0.001
III		0.83	-0.50,2.15			-1.02	-1.63,-0.41	
IV & V		-3.20	-5.25,-1.15			-1.11	-1.89.-0.33	
III *age		-0.16	-0.25,-0.066	0.001		N/A		
IV & V * age		0.50	-0.097,0.20			N/A		

[a]included as time varying covariates; [b] illustrated in Web Figure 1.

P-values for sex interactions: BMI (intercept) P<0.001; BMI (quadratic) P=0.004; BMI (slope) P=0.01; health conditions (slope) P=0.05; smoker P=0.08; sex*age*occupational class P=0.001

Supplementary Table 5. Estimates From Multilevel Models Showing Mean Differences in Grip Strength (kg) by age at First Walking (in 1161men, 2515 observations) and Age at First Standing (in 1211women, 2709 observations) Adjusted for Age Term, Standardised Adult Height, and Then Additionally Adjusted for Each Set of Adult Factors in Turn

	Men: Age at walking (linear)			Men: Age at walking (quadratic)			Women: Age at standing		
	Reg. Coeff.	95% CI	P-value	Reg. Coeff	95% CI	P-value	Reg. Coeff	95% CI	P-value
Adjusted for age, adult height	0.96[b]	-0.62,2.54	0.2	-0.042[b]	-0.096,0.011	0.1	-0.16[b,c]	-0.31,-0.12	0.03
Additional adjustments									
BMI[a], BMI^2, BMI*age	1.02	-0.55,2.60	0.2	-0.044	-0.097,0.0095	0.1	-0.17	-0.32,-0.02	0.03
Health conditions 53y, Health conditions*age	0.99	-0.58,2.57	0.2	-0.044	-0.097,0.0094	0.1	-0.18	-0.33,-0.04	0.01
Educational qualifications	0.88	-0.70,2.46	0.3	-0.040	0.094,0.013	0.1	-0.18	-0.33,-0.03	0.03
Verbal memory[a], Verbal memory*age	0.96	-0.62,2.54	0.2	-0.042	-0.095,0.011	0.1	-0.16	-0.31,-0.01	0.03
Own social class 53y, Own social class*age	1.05	-0.53,2.63	0.2	-0.046	-0.099,0.0072	0.09	-0.16	-0.31,-0.02	0.03
Smoking and exercise[a]	0.90	-0.67,2.47	0.3	-0.041	-0.094,0.012	0.1	-0.17	-0.32,-0.23	0.02

[a]time varying covariates; [b] also adjusted for childhood cognition; [c] also adjusted for birthweight
