# Supplementary material for: Developmental factors associated with decline in grip strength from midlife to old age: a British birth cohort study
Source: BMJ Open. 2019 May 9;9(5):e025755. doi: 10.1136/bmjopen-2018-025755 (PMC6528009; doi:10.1136/bmjopen-2018-025755)
Supplement: Author's manuscript [file bmjopen-2018-025755.draft_revisions.pdf]

BMJ Open is committed to open peer review. As part of this commitment we make the peer review history of every article we publish publicly available.

When an article is published we post the peer reviewers' comments and the authors' responses online. We also post the versions of the paper that were used during peer review. These are the versions that the peer review comments apply to.

The versions of the paper that follow are the versions that were submitted during the peer review process. They are not the versions of record or the final published versions. They should not be cited or distributed as the published version of this manuscript.

BMJ Open is an open access journal and the full, final, typeset and author-corrected version of record of the manuscript is available on our site with no access controls, subscription charges or pay-per-view fees (http://bmjopen.bmj.com).

If you have any questions on BMJ Open's open peer review process please email info.bmjopen@bmj.com

BMJ Open

Developmental factors associated with decline in grip strength from midlife to old age: a British birth cohort study

Journal:	*BMJ Open*
Manuscript ID	bmjopen-2018-025755
Article Type:	Research
Date Submitted by the Author:	31-Jul-2018

Complete List of Authors:	Kuh, Diana; MRC Unit for Lifelong Health and Ageing at UCL,<br>Hardy, Rebecca; University College London<br>Blodgett, Joanna; University College London<br>Cooper, Rachel; UCL, MRC Unit for Lifelong Health and Ageing at UCL

Keywords:	muscle strength, ageing, life course, development, birth cohort

SCHOLARONE™
Manuscripts

Developmental factors associated with decline in grip strength from midlife to old age: a British birth cohort study

Kuh D[1], Hardy R, Blodgett JM[1], Cooper R[1]

[1]MRC Unit for Lifelong Health and Ageing at UCL, London

Abbreviated title: Lifetime risk factors on grip strength decline

Word count:3706

Corresponding author

Professor Diana Kuh PhD FMedSci

MRC Unit for Lifelong Health and Ageing at UCL

33 Bedford Place

London

WC1B 5JU

Tel: + 44 (0) 20 7670 5701

e-mail: d.kuh@ucl.ac.uk

For peer review only

Abstract

Objectives: To test whether developmental factors are associated with grip strength trajectories between 53 and 69 years, and operate independently or on the same pathway/s as adult factors.

Design: British birth cohort study

Setting: England, Scotland and Wales

Participants: 3058 men and women

Main outcome measures: Grip strength (kg) at ages 53, 60-64 and 69, were analysed using multilevel models to estimate associations with developmental factors (birthweight, growth parameters, motor and cognitive development) and father's social class, and investigate adult factors that could explain observed associations, testing for age and sex interactions.

Results: Heavier birthweight, beginning to walk 'on time', later puberty and greater weight 0-26 years in men, and earlier age at first standing in women, were independently associated with stronger grip but not with its decline. The slower decline in grip strength (by 0.068kg/year, 95% confidence interval (CI) 0.024,0.11 per 1SD, p=.003) in men of higher cognitive ability was attenuated by adult verbal memory which became increasingly positively associated with grip strength at older ages.

Conclusions: Patterns of growth and motor development have persisting associations with grip strength between midlife and old age. The strengthening associations with cognition suggest that, at older ages, grip strength increasingly reflects neural ageing processes. Interventions across life that promote muscle development or maintain muscle strength should increase the chance of an independent old age.

For peer review only

Article Summary

Strengths and limitations of this study

- This British birth cohort study has prospectively assessed factors from development onwards, has a wide range of potential covariates, and remains broadly representative of the population born in Britain in the early post war period
- There are repeat measures of grip strength assessed over a relatively long follow-up from midlife (53 years) to early old age (69 years), where there have been few studies of age-related decline.
- It was only possible to model linear change as there are currently only three assessments of grip strength; but we did investigate whether each association strengthened or weakened with age.
- While accounting for deaths and attrition, and those unable to provide a grip measurement for health reasons, did not alter our findings, the observed associations could still be explained by residual confounding.

Key words: muscle strength, ageing, life course trajectory, development, birth cohort

For peer review only

INTRODUCTION

Maintaining musculoskeletal function for the maximal period of time, and preventing musculoskeletal disorders are important aspects of healthy ageing, enabling people to remain active and independent for longer.[1,2] Worldwide, musculoskeletal disorders rank second in years lived with disability and fourth in disability-adjusted life years.[2] The role of muscle mass, strength and metabolic function is recognised for these disorders, and is becoming more widely appreciated in cardiovascular and other chronic diseases.[3,4]

Hand grip strength is a commonly used indicator of muscle strength;[5] and an overall biomarker of ageing.[6-8] Average levels rise to a peak during the early 30s, plateau and then decline.[9-11] Weaker grip is associated with future morbidity, disability and mortality across populations of different ages, ethnicities and income levels,[4,12-18] as is decline in grip strength.[19,20] Adult risk factors, including height and adiposity, health conditions, cognition and health behaviours, have been associated with subsequent grip strength,[21,22] and with age-related decline.[23-34] Developmental factors, such as birthweight, physical growth, motor and cognitive development, and childhood socioeconomic conditions are also related to adult grip strength[35-37] but evidence on whether they are associated with age-related decline is limited.[34,38,39] Using two repeat measures of midlife grip strength in the Medical Research Council (MRC) National Survey of Health and Development (NSHD, the oldest of the British birth cohort studies), Cooper et al. showed that those of higher childhood cognitive ability and SEP (but not higher birthweight) had a reduced risk of decline in grip strength between 53 and 60-64 years.[34] Findings from the Lothian Birth cohort 1921 showed that during late life (79-87 years) those from a lower childhood SEP had a greater decline in grip strength,[39] but there was no association with childhood cognitive ability.[38]

For peer review only

A research gap is to understand whether developmental factors operate on the rate of decline in grip strength independently or on the same pathway/s as adult factors to inform the timing and types of interventions that may modify this decline. Based on previous findings,[34,36,39] and using three repeat measures of grip strength ascertained from age 53 to 69 years in the NSHD, we tested two hypotheses: (1) that higher birthweight, greater gains in height and weight during childhood and adolescence, and earlier puberty are associated with a greater grip strength but not its decline; and (2) that higher childhood cognitive ability, achieving motor milestones early or around the modal age, and higher childhood SEP, are associated with greater grip strength and a slower rate of decline. Further, we expected that any associations between indicators of physical growth and grip strength to be explained by adult health conditions and body mass index (BMI), and that any associations between motor and cognitive development, childhood SEP and grip strength to be explained by education and adult cognition.

METHODS

Sample

The Medical Research Council National Survey of Health and Development (NSHD) is a sample of all births in one week in March 1946 in mainland Britain comprising 5,362 (2,547 female) individuals followed up 24 times, so far to age 69.[40] The maximum sample for these analyses comprised 3,058 participants with at least one measure of grip strength at ages 53, 60-64 or 69 years and known adult height and birthweight. Of the remaining 2,304 in the original birth cohort, 738 had died, 542 were living abroad, 270 had been lost to follow-up, and 166 had not provided all necessary data. Those still participating in the NSHD in adulthood have lower childhood SEP, and higher adult SEP and childhood cognitive ability

For peer review only - http://bmjopen.bmj.com/site/about/guidelines.xhtml

than those who did not, but the sample remains generally representative of those born in Britain in the mid-twentieth century.[41,42] Ethical approval for the most recent visit was given by Queen Square Research Ethics Committee (13/LO/1073) and Scotland A Research Ethics Committee (14/SS/1009). Written informed consent was provided by participants for each visit.

Grip strength

During nurse assessments at ages 53 and 60-64, grip strength was measured in kilograms isometrically using a Nottingham electronic handgrip dynamometer; during a nurse home visit at age 69, a Jamar Plus+ Digital Hand dynamometer was used. A randomised repeated-measurements cross-over trial found no statistically significant differences in values when comparing these two devices.[43] We applied the same standardised protocols and used the maximum of the first four measures (two in each hand) at each age.

Childhood factors

Birthweight: Birthweight, extracted from birth records to the nearest quarter pound, was converted to kilograms.

Physical growth: The SITAR model of growth curve analysis was used to estimate individual patterns of growth in height and weight between 0 and 26 years.[44,45] Heights and weights were measured using standardised protocols at ages 2, 4, 6, 7, 11 and 15, and self-reported at ages 20 and 26. The NSHD data were augmented by height and weight data between 5 and 19 years from the ALSPAC cohort to provide additional information at intermediate ages. Subject-specific random effects were obtained for size, tempo and velocity (in SD units) for height and weight.[44] Later puberty is indicated by positive tempo values and earlier puberty by negative values.[44]

For peer review only

Motor and cognitive development: Age (in months) at first sitting, standing and walking was based on maternal recall at age 2. At age 15, a standardised measure of childhood cognitive ability was derived from the Heim AH4 test of fluid intelligence, the Watts Vernon reading test and a test of mathematical ability.[46,47] Standardised scores from similar tests at ages 11 or 8 were used if missing at age 15 as participants maintained similar ranking across time.

Childhood SEP: Father's occupation at age 4 (or at age 11 or age 15 if missing at age 4), based on the Registrar General's Social Classification, distinguished three groups: high (I or II), middle (IINM or IIM) and low (IV and V).

Adult factors

Height and adiposity: At ages 53, 60-64 and 69, height (cm) and weight (kg) were measured using standard protocols and BMI (kg/m^2) was calculated; standardised scores were used in analyses.

Health conditions: At age 53, a summary of health conditions was a count (0-4) of the presence of knee osteoarthritis, hand osteoarthritis (both based on clinical assessment), severe respiratory symptoms and other potentially disabling or life threatening conditions.[25]

Education and verbal memory: Highest educational attainment by age 26 distinguished those with a degree or higher, advanced secondary, ordinary secondary, other or no qualifications. At ages 53, 60-64 and 69, verbal memory was assessed using a 15 item word list task over three trials (range 0-45),[48] and was converted to a standardised score.

Other adult covariates: At ages 53, 60-64 and 69, participants reported if they smoked and how many times they had taken part in any sports or vigorous leisure activities in the last month (grouped into more than 5 times a month, 1-4 times a month or not at all). Adult SEP, assessed by own occupation at age 53 (or at earlier ages if missing at age 53), distinguished the same three groups as for father's occupation.

For peer review only

Statistical analysis

Stata v14.2 was used for all analyses. We fitted multilevel models which account for the correlation of repeated measures of grip strength within individuals. Preliminary multilevel models tested whether adult height was associated with grip strength and remained constant with age,[33] and whether men had stronger grip but a faster rate of decline than women, as expected.[23,28,29,33,38,49,50]

In the main analysis, models were run separately for men and women because of evidence of sex interactions with age and other covariates; these are reported where $p < 0.1$. All models were adjusted for height, with change in grip strength modelled by a linear age term, and with the intercept and slope fitted as random effects. We also tested for age interactions with each of the risk factors, and for non-linearity with the continuous risk factors (by including a quadratic term). For parsimony, only those age or quadratic terms where $p < 0.1$ remained in the models and are presented in the tables.

To investigate developmental risk factors and grip strength, we first investigated separately the associations with birthweight, physical growth, motor development, cognitive development and childhood SEP. For physical growth, we included all SITAR parameters in the same multilevel models. For motor development, we ran models for age at first sitting, standing and walking, first separately and then mutually adjusted.

Then we took the developmental factors associated with grip strength ($p < 0.05$) at the end of the first stage of the analysis and adjusted in turn for each group of adult factors, having shown in supplementary analyses how these adult factors were associated with grip strength.

For peer review only

In sensitivity analyses, to assess potential bias introduced by: (a) excluding those participants with no valid observations who were unable to perform the test for health reasons, and (b) mortality or other attrition during follow-up, we reran the multilevel models (1) giving a value representing the midpoint of the lowest sex-specific fifth of grip strength to participants unable for health reasons (n=29, 81 observations); and (2) including binary indicators for mortality (n=287) and attrition (n=601).[27,51]

Participant and patient involvement

Participants have a lifelong association with NSHD. Over the 70 years of the study, the research team has increasingly involved participants, in line with changing norms about conducting cohort studies, starting at age 16 (in 1962) with the annual dissemination of study findings in birthday cards. Participants receive personal letters whenever they raise queries or provide additional comments, including suggestions for new topics to study. In the last ten years, the research team has increased the level of participant involvement through invitations to study events (65th and 70th birthday celebrations), public engagement activities, and focus groups to discuss clinical sub-studies. When piloting new questionnaires and assessments, we recruit patients from GP practices or from the UCLH Patient Public Involvement (PPI) group, and take into account their feedback when designing the mainstage fieldwork.

RESULTS

Characteristics of the sample and preliminary analyses

Mean levels of grip strength, birthweight, and adult height were greater in men than women; women had more health conditions and lower SEP than men (Table 1). Mean grip strength declined between ages 53 and 69 by 7.5kg for men and 3.6kg for women; thus the difference

For peer review only - http://bmjopen.bmj.com/site/about/guidelines.xhtml

between men and women attenuated over time, although the mean sex difference remained substantial at age 69 (16.1kg) (Table 1). Preliminary multilevel models confirmed that adult height was strongly associated with grip strength (3.2 kg, 95% confidence interval (CI) 2.8,3.5 per 1SD increase in height, p=<.001) and remained constant with age, and that men had stronger grip and a faster rate of decline than women (p-value<.001 for sex interaction with height).

Developmental risk factors: multilevel models

In models adjusted for adult height and age, there were positive associations between birthweight and grip strength which were stronger in men than women and remained constant with age (Table 2a). In men, having a greater weight between birth and 26 years, and a later puberty (as indicated by a positive coefficient for height tempo) was associated with stronger grip. In women, shorter height, greater weight and a slower weight velocity between birth and 26 years were associated with stronger grip (Table 2b).

Later ages of attaining infant motor milestones of first sitting and standing were associated with weaker grip; there was an inverse U-shaped relationship between age at first walking and grip strength in men (Table 2c-e). These associations remained constant with age. When all three motor milestones, together with height were included together, only the inverse U-shaped relationship between age at first walking and grip strength (for men) and the inverse association between age at first standing and grip strength (for women) remained (Supplementary Table 1); so only these variables were carried forward.

In men, there was an inverse U-shaped relationship between childhood cognitive ability and grip strength at age 53 (Table 2f). The age interaction terms show that the linear term

strengthened (by 0.067kg/year, 95% confidence interval (CI) 0.024, 0.11 per 1SD, P=0.003) and the quadratic term became weaker with age, such that men of higher childhood cognition showed a slower decline in grip strength (Figure 1). In women, higher childhood cognitive ability was associated with stronger grip and this remained constant with age.

There was no association between childhood SEP and grip strength in men (Table 2g). However, in women there was weak evidence that the association grew stronger with age; women from social classes IV and V showed a faster decline in grip strength (Figure 2).

Mutual adjustment of birthweight, physical growth, age at walking (men), age at standing (women), and cognitive development and childhood SEP identified the factors most associated with stronger grip. In men, these were higher birthweight and childhood weight, later puberty, the non-linear relationship with age at first walking and the non-linear relationship with childhood cognition which became increasingly linear with age (Supplementary Table 2). In women, these were earlier age at first standing, and higher childhood cognition; the estimates for the growth parameters were somewhat reduced in this sample with complete childhood data (Supplementary Table 3).

<u>Which adult factors account for the associations between developmental factors and grip strength?</u>

All adult covariates were associated with adult grip strength (supplementary Table 4a-g). Of particular relevance for our hypotheses, higher BMI was associated with stronger grip for men (but not women) and this association levelled off at higher levels of BMI and became weaker with age. Having more health conditions was associated with lower grip strength; for men this association strengthened with age but for women it weakened. Higher educational

For peer review only

levels were associated with stronger grip, especially among women. The association between verbal memory and grip strength was slightly negative in men and not evident in women at age 53 but grew stronger and positive with age (by 0.10kg/year, 95% CI 0.061,0.15 per 1SD, P <0.001) .

In men, the estimates for birthweight, height tempo and weight 0-26 years changed little after adjusting in turn for each adult factor with the greatest reduction in the estimate for birthweight occurring when health conditions were included in the model (Table 3). In contrast, the increasing association between childhood cognitive ability and grip strength with age was strongly attenuated by including verbal memory and a verbal memory by age interaction in the model (Table 4); other adult risk factors had much less impact. The estimate for age at walking was lower in the sample with complete data on adult covariates; however this lower estimate was little affected by adjusting for adult factors (Supplementary Table 5).

In women, the association between childhood cognitive ability and grip strength was reduced by several adult factors, but especially by educational level (Table 5). However, the inverse association between age at first standing and grip strength in women was not reduced by any of the adult factors.

Sensitivity analyses

Giving a value for grip strength representing the mid-point of the lowest sex specific fifth for those who had no measure because of health reasons (maximum of 81 observations) had either no change or marginal change on the estimates. Compared with participants who were followed up and assessed at age 69, those who died during follow-up had lower mean grip strength at age 53 (-2.4kg, 95% CI -4.4,-0.41 for men, P=0.02; and -2.5kg, 95% CI -3.9,-1.2 for women, P<0.001) and, for men, there was weak evidence of a faster decline in grip

For peer review only

strength (by -0.23kg/year, 95% CI -0.37,0.014, P=0.06). The mean differences in grip strength between those dropping out for reasons other than death and those completing follow-up were smaller (-0.99kg, 95%CI -2.4,0.41 for men, P=0.2; and -1.0kg, 95% CI -1.9,-0.16 for women, P=0.02) with no evidence of age interaction. However, estimates for the associations between developmental and adult risk factors and grip strength remained similar after adjusting for death and attrition.

DISCUSSION

In a prospective, nationally representative British birth cohort, developmental indicators had persisting associations with grip strength over 16 years from midlife to old age. Physical growth, in terms of heavier birthweight and later puberty and greater weight throughout the growth period was associated with stronger grip in men, and these effects were robust to adjustment for adult factors. 'On time' motor development for males and advanced motor development for females were associated with stronger grip which were unexplained by adult factors. Childhood cognitive ability was associated with stronger grip (women) and a slower decline in grip strength (men) during that same life stage; these associations were explained by later education, or adult cognition.

Comparisons between studies are difficult because different methods have been used to assess decline in grip strength, not all analyses adjust for current adult height, and few studies have developmental data. Some studies have only two grip assessments,[25,30,33,34,52] or take the difference between the average of several later assessments from the average of several earlier assessments:[32] both methods are limited in their ability to analyse risk factors related to change. Studies with three assessments used bivariate growth curve models[38] or multilevel

For peer review only

models.[27] Other studies with five or more assessments used latent growth curve modelling.[28,53]

In NSHD there was no association between childhood SEP and adult grip strength or its decline after adjusting for developmental factors. Findings from other studies have not been consistent and have adjusted for few, if any, other childhood factors, or have not studied change.[34,36,39,53] The results of a meta-analysis showed modest associations between childhood SEP and adult grip strength at a single time point which were attenuated by adult SEP and current body size, but there was considerable heterogeneity between studies.[54]

The constant effect of birthweight on adult grip strength is consistent with a meta-analysis;[55] this showed a larger estimate for men than women (as this study found) but the sex interaction was not significant. The persistence of the birthweight - grip strength association is worth noting given that more proximal factors may come into play as people age which could have diminished this association; however the size of the estimate suggests that the association may not be clinically meaningful. The persisting associations between growth parameters, motor milestones and grip strength are novel findings, and build on previous NSHD work relating to grip strength at age 53,[36] and bone phenotype at age 60-64.[45,56] Later puberty (in men) was associated with stronger grip, yet earlier puberty was associated with greater areal and volumetric bone mineral density in this cohort,[45] perhaps due to the differential impact of hormonal regulation. Nevertheless, we found that, controlling for contemporaneous body size, greater weight and slower weight velocity throughout the growth period was associated with both greater grip strength and greater bone size,[44] suggesting an extended growth period may benefit both. This could also be the explanation for the persisting associations between motor milestones and grip strength and, in women, for the

For peer review only

inverse association between height during growth and later grip strength, after controlling for adult height.

While the associations between physical growth and grip strength were generally independent of adult covariates, the number of health conditions attenuated the birthweight effect more than other covariates. Lower birthweight is predictive of CVD and diabetes,[57] as is poor muscle strength,[16] and may reflect a common pathway to later disease.

The most striking observations in this study were the strengthening of the positive associations between cognition and grip strength with age, whether cognition was assessed in childhood or adult life. This extends an earlier NSHD study showing that the group with meaningful decline in grip strength between ages 53 and 60-64 had lower childhood cognitive ability than those who experienced no meaningful change.[34] In older cohorts, there is growing evidence that changes in grip strength are related to baseline cognition, and that cognitive decline may precede declines in strength,[24] although the few studies investigating covariation in cognition and grip strength have been inconsistent.[38,58] Our findings complement the findings from older cohorts as they cover midlife changes in grip strength over a longer follow-up period than most previous studies.

A notable implication of our findings regarding lifetime cognition and grip strength is that neural processes have greater impact on grip strength at older ages than in midlife. The attenuation of the childhood cognitive associations once verbal memory or education were taken into account suggests that neurodevelopmental processes play a role in maximising muscle function at maturity but neurodegenerative processes increasingly drive the age-related decline in muscle function. A theoretical model arising out of a review of cognitive

For peer review only

ageing, motor learning and motor skills[59] predicts that ageing impairs cognitive functions before affecting the motor systems and that at older ages the connection between cognition and action becomes stronger, as suggested by our findings. To what extent our findings reflect a direct pathway between brain ageing and muscle strength,[60] or shared mechanisms relating, for example, to haemostatic dysregulation or inflammatory processes,[24,61] is yet to be clarified.

The strengths of NSHD are that it is one of the very few studies with prospectively assessed factors from development onwards, a wide range of potential covariates, and repeat measures of grip strength assessed over a relatively long follow-up period during a critical phase of age-related change. So far these repeat measures cover midlife to early old age, a period which has been studied less often than later ages. NSHD remains broadly representative of the population born in Britain in the early post war period.[42] One limitation is that it is only possible to model linear change as there are currently only three assessments of grip strength. However we did investigate whether each association strengthened or weakened with age. Inevitably there were missing data but neither accounting for deaths and attrition, nor including those unable for health reasons, altered our findings. There were also few differences in the size of the estimates in the models using the maximum samples and the models with complete covariate data. We acknowledge that associations that were maintained after multiple adjustments could be due to residual confounding.

In conclusion, patterns of early growth, attainment of motor milestones, and lifetime cognition have persisting associations with grip strength between midlife and old age, even after taking account of adult body size, health conditions and health behaviours. The impact of neural processes strengthened over this stage of life suggesting that at older ages grip

For peer review only

strength increasingly reflects both physical and cognitive ageing processes. Interventions across life that promote muscle development or maintain peak muscle strength should increase the chance of an active and independent old age.

For peer review only

Table 1. Characteristics of the Sample of 1,528 Men and 1,530 Women in the MRC National Survey of Health and Development With at Least One Measure of Grip Strength at ages 53, 60-64, or 69 and known Height and Birthweight

	Men		Women	
	N	Mean (SD) or %	N	Mean (SD) or %
Grip strength (kg)				
53y	1,398	47.7 (12.2)	1,434	27.7 (7.9)
60-64y	1,003	44.6 (11.6)	1,059	26.0 (7.4)
69y	1,036	40.2 (8.5)	1,062	24.1 (5.8)
Physical growth				
Birthweight (kg)	1,528	3.5 (0.5)	1,530	3.3 (0.5)
Height growth parameters 2-26y	1,509		1,505	
height (cm)		0.095 (6.1)		0.014 (5.8)
height-tempo (%)		-0.079 (6.1)		-0.19 (7.2)
height-velocity (%)		0.23 (10.0)		0.12 (10.6)
Weight growth parameters 0-26y	1,509		1,505	
weight (kg)		-0.25 (4.3)		-0.47 (4.3.)
weight tempo (%)		-0.49 (9.8)		-0.20 (7.7)
weight velocity (%)		-1.6 (26.2)		-0.41 (28.2)
Motor development (months)				
Age at first sitting	1,414	6.6 (1.5)	1,424	6.6 (1.5)
Age at first standing	1,416	11.4 (2.3)	1,419	11.3 (2.1)
Age at first walking	1,424	13.6 (2.5)	1,416	13.6 (2.4)
Early socioeconomic conditions				
Father's occupational class				
I & II	341	23.5	339	23.5
III	707	48.7	719	49.8
IV & V	405	27.9	385	26.7
ADULT FACTORS				
Height (cm)				
53y	1,428	174.7 (6.6)	1,477	161.6 (5.9)
60-64y	1,060	174.8 (6.6)	1,148	161.6 (5.9)
69y	1,038	173.9 (6.4)	1,079	160.6 (6.0)
BMI (kg/m^2)				
53y	1,427	27.4 (4.)	1,466	27.4 (5.4)
63y	1,059	27.9 (4.1)	1,147	27.9 (5.5)
69y	1,038	28.2 (4.6)	1,075	28.2 (5.8)
Verbal memory (no. words)				
53y	1,386	23.0 (6.2)	1,450	24.9 (6.2)
63y	1,020	23.0 (5.9)	1,114	25.4 (6.0)
69y	1,010	21.1 (6.0)	1,057	23.1 (6.0)
No. health conditions 53y				
0	802	56.4	702	47.6
1	448	31.7	543	37.0
2	147	10.5	159	11.3

For peer review only

3+	18	1.4	57	4.1
Smoking status 53y				
Non or ex-smoker	1,094	76.5	1,150	77.7
Smoker	336	23.5	330	22.3
Smoking status 60-64y				
Non or ex-smoker	975	87.2	1,052	88.3
Smoker	136	12.2	140	11.7
Smoking status 69y				
Non or ex-smoker	1,065	89.7	1,140	91.3
Smoker	122	10.2	109	8.7
Leisure-time physical activity 53y				
Inactive	675	47.2	745	50.3
Intermediate	268	18.7	240	16.2
Active	486	34.0	495	33.4
Leisure-time physical activity 60-64y				
Inactive	679	65.2	745	62.9
Intermediate	136	13.0	240	14.4
Active	227	21.8	495	22.7
Leisure-time physical activity 69y				
Inactive	654	60.2	710	60.6
Intermediate	119	11.1	162	13.5
Active	314	28.9	256	25.9
Qualifications by 26y				
Degree or higher	211	14.6	80	5.5
'A-level' or equivalents	410	28.4	338	23.4
'O-level' or equivalents	209	14.5	371	25.7
Less than 'O-level'	90	6.2	133	9.2
None	525	36.3	522	36.1
Own occupational class 53y				
I & II	779	51.6	555	36.6
III	571	37.8	641	42.2
IV & V	161	10.7	322	21.2
Died during follow-up				
No	1,359	88.9	1,412	92.3
Yes	169	11.1	118	7.7
Other attrition during follow-up				
No	1,225	80.2	1,232	80.5
Yes	303	19.8	298	19.5

Standardised score for childhood cognitive ability not presented for 1,408 men and 1,410 women as mean=0 and SD=1

For peer review only

Table 2. Estimates From Multilevel Models Showing Mean Differences in Grip Strength (kg) and Mean Differences in Grip Strength Change (kg/year) for Each Childhood Factor in NSHD. All Models Adjusted for Age Term and Standardised Adult Height.

	MEN				WOMEN			
	N	Reg. Coeff.	95% CI	P-value+	N	Reg. Coeff.	95% CI	P-value+
PHYSICAL GROWTH								
a. Birthweight (kg)	1,528	1.96	1.08,2.84	<0.001	1,530	0.51	-0.07,1.10	0.09
b. Growth parameters	1,509				1,505			
Height -size (cm)		-0.01	-0.22,0.20	0.9		-0.16	-0.29,-0.03	0.02
Height-tempo (%)		0.16	0.06,0.26	0.001		-0.02	-0.04,0.07	0.5
Height-velocity (%)		-0.02	-0.10,0.05	0.5		0.00	-0.04,0.04	0.9
Weight-size (kg)		0.71	0.24,1.18	0.003		0.41	0.57,0.77	0.02
Weight-tempo (%)		-0.06	-0.12,0.01	0.08		-0.01	-0.06,0.04	0.8
Weight-velocity (%)		-0.07	-0.14,0.00	0.06		-0.06	-0.11,0.00	0.04
MOTOR DEVELOPMENT (Months)								
c. Age at sitting	1,414	-0.25	-0.57,0.06	0.1	1,424	-0.18	-0.38,0.01	0.07
d. Age at standing	1,416	-0.27	-0.47,-0.07	0.008	1,419	-0.21	-0.34,-0.07	0.002
e. Age at walking	1,424				1,416			
Walking		1.36	0.00,2.73	0.05		-0.13	-0.25,-0.01	0.03
$Walking^2$		-0.06	-0.10,-0.01	0.01		N/A		
f. COGNITIVE DEVELOPMENT(SD)	1,408				1,410			
Cognition		-0.46	-1.10,0.17	0.1		0.45	0.14,0.76	0.005
$Cognition^2$		-0.81	-1.27,-0.34	0.001		N/A		
Cognition*age (year)		0.07	0.02,0.11	0.003		N/A		
$Cognition^2$*age (year)		0.03	0.00,0.06	0.08		N/A		
g. FATHER'S OCCUPATIONAL CLASS	1,453				1,443			
I & II		REF		0.2		REF		0.4
III		-0.51	-1.64,0.62			-0.24	-1.15,0.67	
IV & V		-1.29	-2.58,0.00			0.38	-0.66,1.42	
III*age (year)		N/A				-0.03	-0.10,0.04	0.07
IV & V*age (year)		N/A				-0.10	-0.18,-0.01	

For peer review only - http://bmjopen.bmj.com/site/about/guidelines.xhtml

+ P-values are given for the overall tests of associations. P-values for sex interactions: birthweight p=.004; childhood height after adjustment for adult height p-value .07; height tempo p=.006; age at first waking (quadratic term) p=.02

For peer review only

Table 3. Estimates From Multilevel Models Showing Mean Differences in Grip Strength (kg) by Birthweight, Height Tempo and Weight-Size in 1,295 NSHD Men (2,788 Observations), Adjusted for Age Term, Standardised Adult Height and all Growth Parameters, and Then Additionally Adjusted for Each Set of Adult Factors in Turn

	Birthweight (kg)			Height Tempo (%)			Weight-size (kg)		
	Reg. Coeff.	95% CI	P-value	Reg. Coeff.	95% CI	P-value	Reg. Coeff.	95% CI	P-value
Adjusted for age, all growth parameters and adult height	1.35	0.33,2.37	0.009	0.25	0.14,0.35	<0.001	0.63	0.10,1.16	0.02
Additional adjustments in turn									
BMI[a], BMI^2, BMI*age	1.37	0.35,2.38	0.008	0.25	0.14,0.35	<0.001	0.60	0.07,1.12	0.03
Health conditions 53y Health conditions*age	1.28	0.27,2.29	0.01	0.24	0.13,0.35	<0.001	0.60	0.07,1.12	0.03
Qualifications	1.42	0.40,2.43	0.006	0.25	0.14,0.36	<0.001	0.61	0.08,1.14	0.02
Verbal memory[a], Verbal memory*age	1.39	0.37,2.41	0.008	0.24	0.13,0.35	<0.001	0.62	0.09,1.15	0.02
Own social class 53y, own social class*age	1.42	0.40,2.43	0.006	0.24	0.14,0.35	<0.001	0.60	0.07,1.12	0.03
Smoking & physical activity[a]	1.39	0.38,2.40	0.007	0.24	0.13,0.34	<0.001	0.63	0.10,1.15	0.02

For peer review only

Table 4. Estimates From Multilevel Models Showing Mean Differences in Grip Strength (kg) and Mean Differences in Grip Strength Change (kg/year) by Childhood Cognition in 1,161 NSHD Men (2,515 Observations), Adjusted for Age Term, Standardised Adult height and Age at First Walking, and Then Additionally Adjusted for Each Set of Adult Factors in Turn

	Childhood cognition (SD)			Childhood cognition $(SD)^2$			Childhood cognition (SD)*age (year)		
	Reg. Coeff.	95% CI	P-value	Reg. Coeff.	95% CI	P-value	Reg. Coeff.	95% CI	P-value
Adjusted for age, adult height, walking, $walking^2$	-0.44	-1.14,0.25	0.2	-0.52	-0.94,-0.11	0.01	0.08	0.028,0.13	0.002
Additional adjustments in turn									
BMI[a], BMI^2, BMI*age	-0.37	-1.06,0.32	0.3	-0.51	-0.93,-0.10	0.01	0.07	0.02,0.12	0.005
Health conditions 53y Health conditions*age	-0.55	-1.25,0.15	0.1	-0.53	-0.95,-0.12	0.01	0.08	0.03,0.13	0.002
Qualifications	-0.79	-1.62,0.04	0.06	-0.56	-0.98,0.14	0.009	0.08	0.03,0.13	0.002
Verbal memory [a], verbal memory*age	-0.12	-0.93,0.69	0.7	-0.54	-0.96,-0.12	0.01	0.03	-0.03.0.09	0.3
Own social class 53y, own social class*age	-0.80	-1.59,0.00	0.05	-0.52	-0.94,-0.10	0.01	0.07	-0.02,0.13	0.01
Smoking & physical activity[a]	-0.64	-1.34,0.06	0.07	-0.56	-0.97,-0.14	0.08	0.08	0.03,0.13	0.001

[a]time varying covariates;

For peer review only

Table 5. Estimates From Multilevel Models Showing Mean Differences in Grip Strength (kg) by Age at First Standing and Childhood Cognition in 1,211 NSHD Women (2,709 Observations) , Adjusted for Age Term and Standardised Adult Height, and Then Additionally Adjusted for Each Set of Adult Factors in Turn.

	Age at first standing (months)			Childhood cognition (SD)		
	Reg. Coeff.	95% CI	P-value	Reg. Coeff.	95% CI	P-value
Adjusted for age and adult height, and mutually adjusted	-0.16	-0.31,-0.12	0.03	0.37	0.031,0.71	0.03
Additional adjustments in turn						
BMI[a]	-0.17	-0.32,-0.02	0.03	0.36	0.02,0.70	0.04
Health conditions 53y[a], health conditions*age	-0.18	-0.33,-0.04	0.01	0.27	-0.07,0.61	0.1
Qualifications[a]	-0.18	-0.33,-0.03	0.03	0.019	-0.4,0.48	0.9
Verbal memory[a], verbal memory*age	-0.16	-0.31,-0.01	0.03	0.22	-0.17,0.61	0.3
Own social class	-0.16	-0.31,-0.02	0.03	0.27	-0.10,0.65	0.1
Smoking & physical activity[a]	-0.17	-0.32,-0.23	0.02	0.30	-0.04,0.65	0.08

[a] time varying covariates;

For peer review only

ACKNOWLEDGEMENTS

We thank NSHD study members for their lifelong participation and past and present members of the NSHD study team who helped to collect the data.

FUNDING

This work was supported by the UK Medical Research Council MC_UU_12019/1 which provides core funding for the MRC National Survey of Health and Development and supports DK, JB, RC, and RH by MC_UU_12019/1, MC_UU_12019/2, MC_UU_12019/4. JB also receives support from UCL (Overseas and Graduate Research Scholarships). The funders had no role in the study or the decision to submit the paper for publication.

AUTHOR CONTRIBUTIONS

DK, RC, JB and RH contributed to the study design and data interpretation and DK, RC and RH collected the data. DK undertook the literature search, the data analysis and wrote the first draft of the manuscript; all authors revised the manuscript. DK is the guarantor and accepts full responsibility for the work and the conduct of the study, had access to the data, and controlled the decision to publish. All authors, external and internal, had full access to all of the data (including statistical reports and tables) in the study and can take responsibility for the integrity of the data and the accuracy of the data analysis.

OPEN ACCESS: This is an Open Access article distributed in accordance with the terms of the Creative Commons Attribution (CC BY 3.0) license, which permits others to distribute, remix, adapt and build upon this work, for commercial use, provided the original work is properly cited..

For peer review only

DECLARATION OF COMPETING INTERESTS: All authors have completed the ICMJE uniform disclosure form and declare: DK, JB, RC, and RH received financial support from the UK Medical Research Council for the submitted work. JB also receives support from UCL (Overseas and Graduate Research Scholarships); no financial relationships with any organisations that might have an interest in the submitted work in the previous three years; no other relationships or activities that could appear to have influenced the submitted work.

DATA SHARING STATEMENT: Data used in this publication are available to bona fide researchers upon request to the NSHD Data Sharing Committee via a standard application procedure. Further details can be found at http://www.nshd.mrc.ac.uk/data. doi:10.5522/NSHD/Q101; doi:10.5522/NSHD/Q102; 10.5522/NSHD/Q103

For peer review only

Figure Legends

Figure 1. Mean grip strength (kg) by childhood cognition for men of mean height (based on 1408 men)

Figure 2. Mean grip strength (kg) by father's social class for women of mean height (based on 1443 women)

References

1. Kuh D, Karunananthan S, Bergman H, Cooper R. A life-course approach to healthy ageing: maintaining physical capability. *Proc Nutr Soc* 2014; **73**(2): 237-48.
2. Briggs AM, Cross MJ, Hoy DG, et al. Musculoskeletal Health Conditions Represent a Global Threat to Healthy Aging: A Report for the 2015 World Health Organization World Report on Ageing and Health. *Gerontologist* 2016; 56 Suppl 2: S243-55.
3. Wolfe RR. The underappreciated role of muscle in health and disease. *Am J Clin Nutr* 2006; **84**(3): 475-82.
4. Manring H, Abreu E, Brotto L, Weisleder N, Brotto M. Novel excitation-contraction coupling related genes reveal aspects of muscle weakness beyond atrophy-new hopes for treatment of musculoskeletal diseases. *Frontiers in Physiol* 2014; **5**: 37.
5. Bohannon RW. Are hand-grip and knee extension strength reflective of a common construct? *Percept Mot Skills* 2012; **114**(2): 514-8.
6. Martin-Ruiz CM, von Zglinicki T. A life course approach to biomarkers of ageing. In: Kuh D, Cooper R, Hardy R, Richards M, Ben-Shlomo Y, eds. A life course approach to healthy ageing. 1st ed. Oxford: Oxford University Press; 2014: 177-86.
7. Sayer AA, Kirkwood TB. Grip strength and mortality: a biomarker of ageing? *Lancet* 2015; **386**(9990): 226-7.
8. Justice JN, Cesari M, Seals DR, Shively CA, Carter CS. Comparative Approaches to Understanding the Relation Between Aging and Physical Function. *J Gerontol A Biol Sci Med Sci* 2016; **71**(10): 1243-53.
9. Dodds RM, Syddall HE, Cooper R, et al. Grip strength across the life course: normative data from twelve British studies. *PLoS ONE* 2014; **9**(12): e113637.
10. Dodds RM, Syddall HE, Cooper R, Kuh D, Cooper C, Sayer AA. Global variation in grip strength: a systematic review and meta-analysis of normative data. *Age Ageing* 2016; **45**(2): 209-16.
11. Nahhas RW, Choh AC, Lee M, et al. Bayesian longitudinal plateau model of adult grip strength. *Am J Hum Biol* 2010; **22**(5): 648-56.
12. Rantanen T, Volpato S, Ferrucci L, Heikkinen E, Fried LP, Guralnik JM. Handgrip strength and cause-specific and total mortality in older disabled women: exploring the mechanism. *JAm Geriatr Soc* 2003; **51**(5): 636-41.

For peer review only

13. Cooper R, Kuh D, Hardy R. Objectively measured physical capability levels and mortality: systematic review and meta-analysis. *BMJ* 2010; **341**: c4467.
14. Cooper R, Kuh D, Cooper C, et al. Objective measures of physical capability and subsequent health: a systematic review. *Age Ageing* 2011; **40**: 14-23.
15. den Ouden ME, Schuurmans MJ, Arts IE, van der Schouw YT. Physical performance characteristics related to disability in older persons: a systematic review. *Maturitas* 2011; **69**(3): 208-19.
16. Leong DP, Teo KK, Rangarajan S, et al. Prognostic value of grip strength: findings from the Prospective Urban Rural Epidemiology (PURE) study. *Lancet* 2015; **386**(9990): 266-73.
17. Ortega FB, Silventoinen K, Tynelius P, Rasmussen F. Muscular strength in male adolescents and premature death: cohort study of one million participants. *BMJ* 2012; **345**: e7279.
18. Kim Y, Wijndaele K, Lee DC, Sharp SJ, Wareham N, Brage S. Independent and joint associations of grip strength and adiposity with all-cause and cardiovascular disease mortality in 403,199 adults: the UK Biobank study. *Am J Clin Nutr* 2017; **106**(3): 773-82.
19. Peterson MD, Zhang P, Duchowny KA, Markides KS, Ottenbacher KJ, Snih SA. Declines in Strength and Mortality Risk Among Older Mexican Americans: Joint Modeling of Survival and Longitudinal Data. *J Gerontol A Biol Sci Med Sci* 2016; **71**(12): 1646-52.
20. Syddall HE, Westbury LD, Dodds R, Dennison E, Cooper C, Sayer AA. Mortality in the Hertfordshire Ageing Study: association with level and loss of hand grip strength in later life. *Age Ageing* 2017; **46**(3): 407-12.
21. Shaw SC, Dennison EM, Cooper C. Epidemiology of Sarcopenia: Determinants Throughout the Lifecourse. *Calcif Tissue Int* 2017; **101**(3): 229-47.
22. Clouston SAP, Brewster P, Kuh D, et al. The dynamic relationship between physical function and cognition in longitudinal aging cohorts. *Epidemiologic Reviews* 2013; **35**(1): 33-50.
23. Syddall HE, Westbury LD, Shaw SC, Dennison EM, Cooper C, Gale CR. Correlates of Level and Loss of Grip Strength in Later Life: Findings from the English Longitudinal Study of Ageing and the Hertfordshire Cohort Study. *Calcif Tissue Int* 2017.
24. Fritz NE, McCarthy CJ, Adamo DE. Handgrip strength as a means of monitoring progression of cognitive decline - A scoping review. *Ageing ResRev* 2017; **35**: 112-23.
25. Cooper R, Muniz-Terrera G, Kuh D. Associations of behavioural risk factors and health status with changes in physical capability over 10 years of follow-up: the MRC National Survey of Health and Development. *BMJ Open* 2016; **6**(4): e009962.
26. Granic A, Davies K, Jagger C, Kirkwood TB, Syddall HE, Sayer AA. Grip Strength Decline and Its Determinants in the Very Old: Longitudinal Findings from the Newcastle 85+ Study. *PLoSOne* 2016; **11**(9): e0163183.
27. Botoseneanu A, Bennett JM, Nyquist L, et al. Cardiometabolic Risk, Socio-Psychological Factors, and Trajectory of Grip Strength Among Older Japanese Adults. *JAging Health* 2015; **27**(7): 1123-46.
28. Sternang O, Reynolds CA, Finkel D, Ernsth-Bravell M, Pedersen NL, Dahl Aslan AK. Factors associated with grip strength decline in older adults. *Age Ageing* 2015; **44**(2): 269-74.
29. Stenholm S, Tiainen K, Rantanen T, et al. Long-Term Determinants of Muscle Strength Decline: Prospective Evidence from the 22-Year Mini-Finland Follow-Up Survey. *J Am Geriatr Soc* 2012; **60**(1): 77-85.
30. Miller DK, Malmstrom TK, Miller JP, Andresen EM, Schootman M, Wolinsky FD. Predictors of change in grip strength over 3 years in the African American health project. *J Aging Health* 2010; **22**(2): 183-96.

For peer review only

31. Rantanen T, Penninx BW, Masaki K, Lintunen T, Foley D, Guralnik JM. Depressed mood and body mass index as predictors of muscle strength decline in old men. *J Am Geriatr Soc* 2000; **48**(6): 613-7.
32. Rantanen T, Masaki K, Foley D, Izmirlian G, White L, Guralnik JM. Grip strength changes over 27 yr in Japanese-American men. *J Applied Physiol* 1998; **85**(6): 2047-53.
33. Clement FJ. Longitudinal and cross-sectional assessments of age changes in physical strength as related to sex, social class, and mental ability. *J Gerontol* 1974; **29**: 423-9.
34. Cooper R, Richards M, Kuh D. Childhood Cognitive Ability and Age-Related Changes in Physical Capability From Midlife: Findings From a British Birth Cohort Study. 2017; **79**(7): 785-91.
35. Aihie Sayer A, Cooper C, Evans JR, et al. Are rates of ageing determined in utero? *Age Ageing* 1998; **27**: 579-83.
36. Kuh D, Hardy R, Butterworth S, et al. Developmental origins of midlife grip strength: Findings from a birth cohort study. *J Gerontol Series A-Biol Sci Med Scis* 2006; **61**(7): 702-6.
37. Cooper R, Hardy R, Sayers A, Kuh D. A life course approach to physical capability. In: Kuh D, Cooper R, Hardy R, Richards M, Ben-Shlomo Y, eds. A life course approach to healthy ageing. 1st ed. Oxford: Oxford University Press; 2014: 16-31.
38. Deary IJ, Johnson W, Gow AJ, et al. Losing one's grip: a bivariate growth curve model of grip strength and nonverbal reasoning from age 79 to 87 years in the Lothian Birth Cohort 1921. *J Gerontol B Psychol Sci Soc Sci* 2011; **66**(6): 699-707.
39. Starr JM, Deary IJ. Socio-economic position predicts grip strength and its decline between 79 and 87 years: the Lothian Birth Cohort 1921. *Age and Ageing* 2011; **40**(6): 749-52.
40. Kuh D, Wong A, Shah I, et al. The MRC National Survey of Health and Development reaches age 70: maintaining participation at older ages in a birth cohort study. *Eur J Epidemiol* 2016; **31**(11): 1135-47.
41. Wadsworth ME, Butterworth SL, Hardy RJ, et al. The life course prospective design: an example of benefits and problems associated with study longevity. *Soc Sci Med* 2003; **57**(11): 2193-205.
42. Stafford M, Black S, Shah I, et al. Using a birth cohort to study ageing: representativeness and response rates in the National Survey of Health and Development. *Eur J Ageing* 2013; **10**(2): 145-57.
43. Lessof C. Testing for differences in measurement devices: findings from a randomized trial to compare measures of physiological function and physical performance European Survey Research Association; 2017 19th July; Lisbon; 2017.
44. Cole TJ, Kuh D, Johnson W, et al. Using Super-Imposition by Translation And Rotation (SITAR) to relate pubertal growth to bone health in later life: the Medical Research Council (MRC) National Survey of Health and Development. *Int J Epidemiol* 2016; **45**(4): 1125-34.
45. Kuh D, Muthuri SG, Moore A, et al. Pubertal timing and bone phenotype in early old age: findings from a British birth cohort study. *Int J Epidemiol* 2016; **45**(4): 1113-24.
46. Heim AW. The AH4 group test of intelligence. Windsor: NFER-Nelson; 1970.
47. Pigeon DA. Details of the fifteen years tests. In: JWB D, JM R, HR S, eds. All our future. London: Davies; 1968: Appendix 1.
48. Davis D, Bendayan R, Muniz G, R H, M R, Kuh. Decline in search speed and verbal memory over 26 years of midlife in a British birth cohort. *Neuroepidemiol* 2017;**49** (3-4):121-8.
49. Oksuzyan A, Maier H, McGue M, Vaupel JW, Christensen K. Sex Differences in the Level and Rate of Change of Physical Function and Grip Strength in the Danish 1905-Cohort Study. *J Aging Health* 2010; **22**(5): 589-610.

For peer review only

50. Botoseneanu A, Allore HG, Mendes de Leon CF, Gahbauer EA, Gill TM. Sex Differences in Concomitant Trajectories of Self-Reported Disability and Measured Physical Capacity in Older Adults. *J Gerontol A Biol Sci Med Sci* 2016; **71**(8): 1056-62.
51. Botoseneanu A, Liang J. The effect of stability and change in health behaviors on trajectories of body mass index in older Americans: a 14-year longitudinal study. *J Gerontol A Biol Sci Med Sci* 2012; **67**(10): 1075-84.
52. Daly RM, Ahlborg HG, Ringsberg K, Gardsell P, Sernbo I, Karlsson MK. Association between changes in habitual physical activity and changes in bone density, muscle strength, and functional performance in elderly men and women. *J Am Geriatr Soc* 2008; **56**(12): 2252-60.
53. Kroger H, Fritzell J, Hoffmann R. The Association of Levels of and Decline in Grip Strength in Old Age with Trajectories of Life Course Occupational Position. *PLoSOne* 2016; **11**(5): e0155954.
54. Birnie K, Cooper R, Martin RM, et al. Childhood socioeconomic position and objectively measured physical capability levels in adulthood: A systematic review and meta-analysis. *PLoS ONE* 2011; **6**(1): e15564.
55. Dodds R, Denison HJ, Ntani G, et al. Birth weight and muscle strength: A systematic review and meta-analysis. *J Nutr Health Ageing* 2012; **16**(7): 609-15.
56. Kuh D, Wills AK, Shah I, et al. Growth from birth to adulthood and bone phenotype in early old age: A British birth cohort study. *J Bone Miner Res* 2014; **29**(1): 123-33.
57. Whincup PH, Kaye SJ, Owen CG, et al. Birth weight and risk of type 2 diabetes: A systematic review. *JAMA* 2008; **300**(24): 2886-97.
58. Praetorius Bjork M, Johansson B, Hassing LB. I forgot when I lost my grip-strong associations between cognition and grip strength in level of performance and change across time in relation to impending death. *Neurobiol aging* 2016; **38**: 68-72.
59. Ren J, Wu YD, Chan JS, Yan JH. Cognitive aging affects motor performance and learning. *Geriatr Gerontol Int* 2013; **13**(1): 19-27.
60. Bhanushali M, Conwit R, Metter RM, Ferrucci L. The role of the nervous system in muscle atrophy. In: Cruz-Jentoft AJ,Morley JE, editors. Sarcopenia. Chichester, UK: John Wiley & Sons; 2012.
61. Newman AB, Sanders JL, Kizer JR, et al. Trajectories of function and biomarkers with age: the CHS All Stars Study. *Int J Epidemiol* 2016; **45**(4): 1135-45.

For peer review only

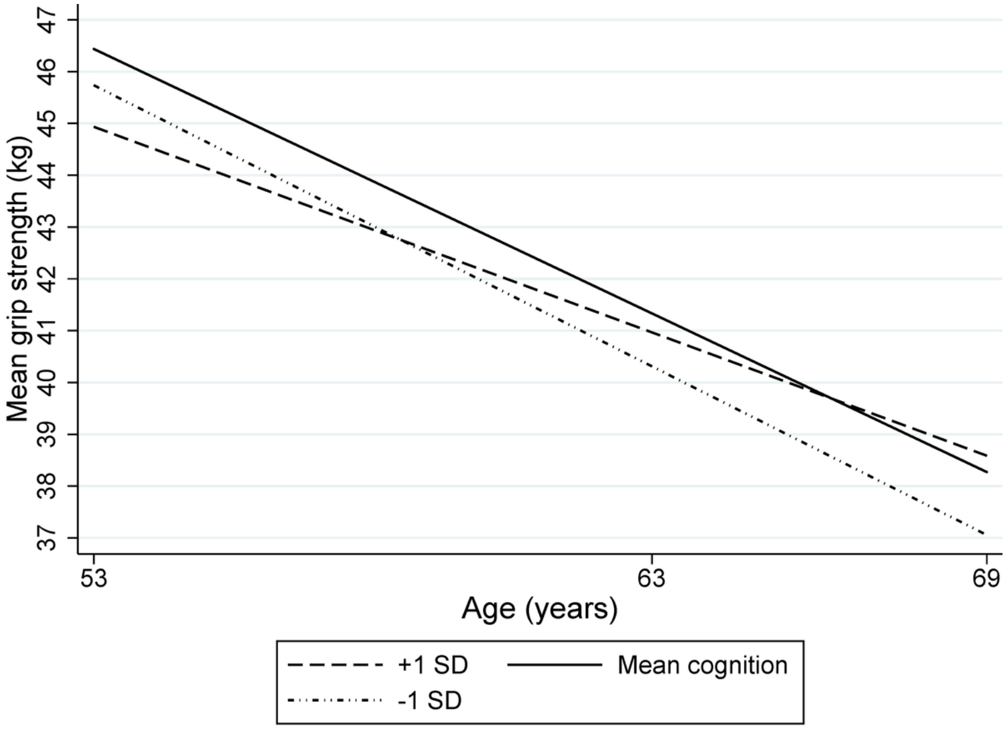

Figure 1. Mean grip strength (kg) by childhood cognition for men of mean height (based on 1408 men)

101x73mm (300 x 300 DPI)

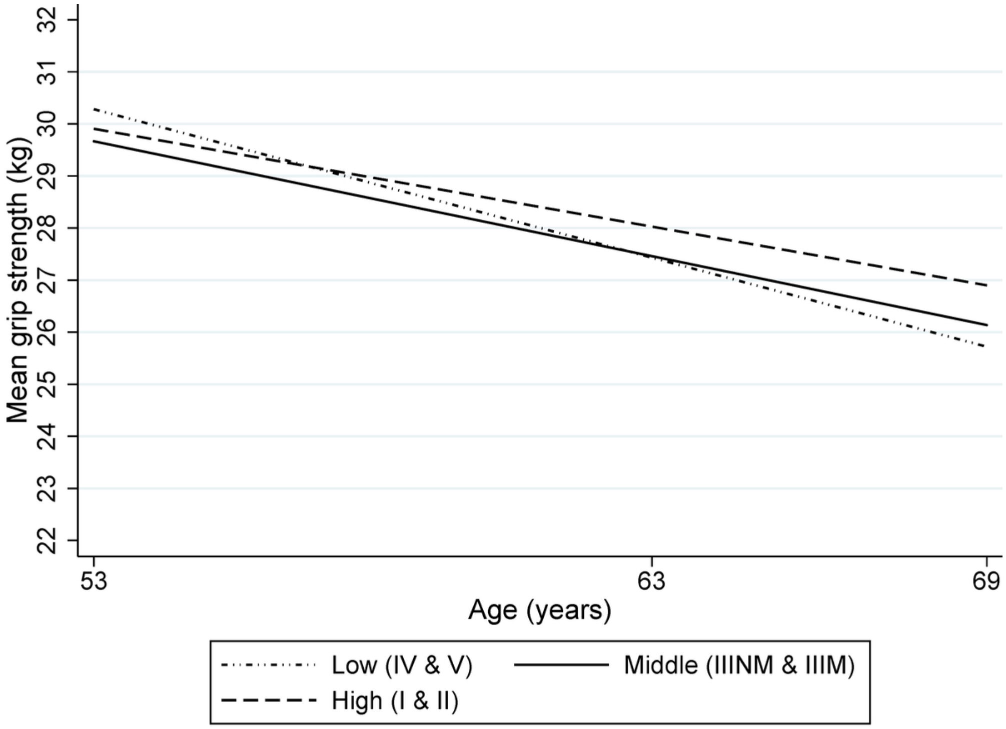

Figure 2. Mean grip strength (kg) by father’s social class for women of mean height (based on 1443 women)

101x73mm (300 x 300 DPI)

1

Developmental factors associated with decline in grip strength from midlife to old age: a British birth cohort study

Kuh D[1], Hardy R, Blodgett JM[1], Cooper R[1]

[1]MRC Unit for Lifelong Health and Ageing at UCL, London

SUPPLEMENTARY TABLES

For peer review only

For peer review only - http://bmjopen.bmj.com/site/about/guidelines.xhtml

Supplementary Table 1. Estimates From Multilevel Models Showing Mean Differences in Grip Strength (kg) in 1,375 NSHD Men (3,111 Observations) and in 1,385 NSHD Women (3,266 Observations) for Mutually Adjusted Physical Growth Indicators. All Models Adjusted for Age Term and Adult height and Using Sample With Complete Childhood Data.

	MEN			WOMEN		
	Reg. Coeff.	95% CI	P-value	Reg. Coeff.	95% CI	P-value
Age (months) at sitting	0.030	-0.35,0.40	0.9	-0.031	-0.27,0.21	0.8
Age (months) at standing	0.14	-0.23,0.46	0.6	-0.29	-0.53,-0.052	0.02
Age (months) at walking						
Walking	1.72	0.33,3.1	0.01	0.11	-0.093,0.32	0.3
$Walking^2$	-0.075	-0.12,-0.028	0.002	N/A		

For peer review only - http://bmjopen.bmj.com/site/about/guidelines.xhtml

Supplementary Table 2. Estimates From Multilevel Models Showing Mean Differences in Grip Strength (kg) and Mean Differences in Grip Strength Change (kg/year) in 1,316 NSHD Men (2,983 Observations) for Mutually Adjusted Childhood Factors. All Models Adjusted for Age Term and Standardised Adult Height.

	Adjusted for age term and height			Fully adjusted		
	Reg. Coeff.	95% CI	P-value+	Reg. Coeff.	95% CI	P-value+
BIRTHWEIGHT (kg)	2.00	1.05,2.95	<0.001	1.40	0.41,2.38	0.005
GROWTH PARAMETERS						
Height -size (cm)	-0.09	-0.32,0.14	0.4	-0.07	-0.30,0.16	0.5
Height-tempo (%)	0.16	0.052,0,26	0.004	0.15	0.046,0.26	0.005
Height-velocity (%)	-0.02	-0.10,0.06	0.6	-0.03	-0.10,0.05	0.5
Weight-size (kg)	0.93	0.41,1.44	<0.001	0.62	0.094,1.15	0.02
Weight-tempo (%)	-0.07	-0.13,0.00	0.051	-0.05	-0.12,0.02	0.1
Weight-velocity (%)	-0.10	-0.18,-0.02	0.011	-6.16	-14.11,1.79	0.1
MOTOR DEVELOPMENT (months)						
Age at walking						
Walking	1.35	-0.073,2.78	0.06	1.30	-0.11,2.72	0.07
Walking^2	-0.057	-0.10,-0.01	0.02	-0.054	-0.10,-0.01	0.02
CHILDHOOD DEVELOPMENT (SD)						
Cognition	-0.32	-0.98,0.33	0.3	-0.41	-1.08,0.27	0.2
Cognition^2	-0.78	-1.25,-0.30	0.001	-0.77	-1.24,-0.30	0.001
Cognition*age (year)	0.07	0.021,0.11	0.004	0.067	0.02,0.11	0.003
Cognition^2 * age (year)	0.03	-0.01,0.06	0.09	0.024	-0.01,0.06	0.2
FATHER'S OCCUPATIONAL CLASS						
I & II	REF		0.08	REF		0.09
III	-0.62	-1.81,0.58		-0.77	-1.99,0.44	
IV and V	-1.55	-2.91,-0.18		-1.63	-3.08,-0.18	

+ P-values are given for the overall tests of associations

For peer review only - http://bmjopen.bmj.com/site/about/guidelines.xhtml

Supplementary Table 3. Estimates From Multilevel Models Showing Mean Differences in Grip Strength (kg) and Mean Differences in Grip Strength Change (kg/year) in 1,320 NSHD Women (3,069 observations) for Mutually Adjusted Childhood Factors. All Models Adjusted for Age Term and Standardised Adult Height.

	Adjusted for age term and height			Fully adjusted		
	Reg. Coeff.	95% CI	P-value+	Reg. Coeff.	95% CI	P-value+
BIRTHWEIGHT (kg)	0.57	-0.062,1.20	0.08	0.34	-0.31,0.98	0.3
GROWTH PARAMETERS						
Height -size (cm)	-0.14	-0.28,0.01	0.07	-0.14	-0.29,-0.01	0.06
Height-tempo (%)	0.02	-0.041,0.08	0.6	0.02	-0.04,0.07	0.6
Height-velocity (%)	-0.01	-0.05,0.037	0.7	-0.01	-0.05,0.04	0.8
Weight-size (kg)	0.36	-0.02,0.75	0.06	0.31	-0.08,0.71	0.1
Weight-tempo (%)	0.00	-0.05,0.05	1.0	0.01	-0.04,0.06	0.8
Weight-velocity (%)	-0.05	-0.11,0.07	0.08	-0.04	-0.99,0.15	0.1
MOTOR DEVELOPMENT (months)						
Age at standing	-0.16	2.50,3.39	0.03	-0.15	-0.29,0.00	0.05
CHILDHOOD DEVELOPMENT (SD)						
Cognition	0.41	0.09,0.73	.01	0.40	0.053,0.74	0.02
FATHER'S OCCUPATIONAL CLASS						
I & II	REF		0.2	REF		0.2
III	-0.59	-1.65,0.47		-0.45	-1.53,0.62	
IV and V	0.22	-0.99,1.43		0.52	-0.72,1.76	
III *age (year)	-0.02	-0.10,0.06	0.2	-0.02	-0.97,0.06	0.1
IV and V*age (year)	-0.08	-0.17,0.01		-0.08	-0.17,0.01	

+ P-values are given for the overall tests of associations

For peer review only - http://bmjopen.bmj.com/site/about/guidelines.xhtml

Supplementary Table 4 Estimates From Multilevel Models Showing Mean Differences in Grip Strength (kg) and Mean Differences in Grip Strength Change (kg/year) by Each Adult Factor. All Models Adjusted for Age Term and Standardised Adult Height.

		Men					Women	
	n	Reg. Coeff.	95% CI	P-value	n	Reg. Coeff.	95% CI	P-value
a.BMI (per SD)[a]	1,528				1,524			
BMI		1.66	0.93,2.38	<0.001		-0.18	-0.42,0.049	0.1
BMI^2		-0.49	-0.80,-0.18	0.002		n/a		
BMI*age		-0.057	-0.11,-0.0056	0.03		n/a		
b. Health conditions at 53y	1,415				1,441			
Health conditions		-0.84	-1.70,0.19	0.05		-1.42	-1.89,-0.94	<0.001
Health conditions^2		-0.055	-0.12,0.0057	0.08		0.030	-0.0066,0.066	0.1
c. Smoker v. non-smoker [a]	1,516	-1.28	-2.38,-0.18	0.02	1,520	-0.17	-0.90,0.55	0.6
d. Leisure time physical activity[a]	1,521					1522		
Inactive		REF		0.003		REF		0.005
Intermediate		1.52	0.54,2.51			0.85	0.21,1.48	
Active		1.04	0.23,1.84			0.71	0.18,1.24	
e. Verbal memory[a]	1,489				1,508			
Verbal memory		-0.67	-1.24,-0.093	0.02		0.070	-0.31,0.45	0.7
Verbal memory*age		0.10	0.061,0.15	<0.001		0.032	0.0020,0.063	0.04
f. Qualifications (per step)	1,445	0.17	-0.13,0.48	0.3	1,444	0.38	0.16,0.59	0.001
g. Own occupational class at 53y[b]	1,511				1,518			
I and II		REF		0.001		REF		0.001
III		0.83	-0.50,2.15			-1.02	-1.63,-0.41	
IV & V		-3.20	-5.25,-1.15			-1.11	-1.89.-0.33	
III *age		-0.16	-0.25,-0.066	0.001		N/A		
IV & V * age		0.50	-0.097,0.20			N/A		

[a]included as time varying covariates; [b] illustrated in Web Figure 1.

For peer review only - http://bmjopen.bmj.com/site/about/guidelines.xhtml

6

P-values for sex interactions: BMI (intercept) P<0.001; BMI (quadratic) P=0.004; BMI (slope) P=0.01; health conditions (slope) P=0.05; smoker P=0.08; sex*age*occupational class P=0.001

For peer review only

For peer review only - http://bmjopen.bmj.com/site/about/guidelines.xhtml

Supplementary Table 5. Estimates From Multilevel Models Showing Mean Differences in Grip Strength (kg) by age at First Walking, Adjusted for Age Term, Standardised Adult Height and Childhood Cognition in 1,161 NSHD men (2,515 Observations), and Then Additionally Adjusted for Each Set of Adult Factors in Turn

	Men: Age at walking (linear)			Men: Age at walking (quadratic)		
	Reg. Coeff.	95% CI	P-value	Reg. Coeff	95% CI	P-value
Adjusted for age, adult height	0.96[b]	-0.62,2.54	0.2	-0.042[b]	-0.096,0.011	0.1
Additional adjustments						
BMI[a], BMI^2, BMI*age	1.02	-0.55,2.60	0.2	-0.044	-0.097,0.0095	0.1
Health conditions 53y, Health conditions*age	0.99	-0.58,2.57	0.2	-0.044	-0.097,0.0094	0.1
Educational qualifications	0.88	-0.70,2.46	0.3	-0.040	0.094,0.013	0.1
Verbal memory[a], Verbal memory*age	0.96	-0.62,2.54	0.2	-0.042	-0.095,0.011	0.1
Own occupational class 53y, Own occupational class*age	1.05	-0.53,2.63	0.2	-0.046	-0.099,0.0072	0.09
Smoking and exercise[a]	0.90	-0.67,2.47	0.3	-0.041	-0.094,0.012	0.1

[a]time varying covariates; [b] also adjusted for childhood cognition; [c] also adjusted for birthweight

For peer review only

For peer review only - http://bmjopen.bmj.com/site/about/guidelines.xhtml

STROBE 2007 (v4) Statement—Checklist of items that should be included in reports of *cohort studies*

Checklist completed for: Developmental and adult risk factors associated with decline in grip strength from midlife to old age: a British birth cohort study

Section/Topic	Item #	Recommendation	Reported on page #
**Title and abstract**	1	(*a*) Indicate the study's design with a commonly used term in the title or the abstract	Title & abstract
		(*b*) Provide in the abstract an informative and balanced summary of what was done and what was found	2
**Introduction**			
Background/rationale	2	Explain the scientific background and rationale for the investigation being reported	4-5
Objectives	3	State specific objectives, including any prespecified hypotheses	5
**Methods**			
Study design	4	Present key elements of study design early in the paper	5-7
Setting	5	Describe the setting, locations, and relevant dates, including periods of recruitment, exposure, follow-up, and data collection	5-7
Participants	6	(*a*) Give the eligibility criteria, and the sources and methods of selection of participants. Describe methods of follow-up	5-7
		(*b*) For matched studies, give matching criteria and number of exposed and unexposed	n/a
Variables	7	Clearly define all outcomes, exposures, predictors, potential confounders, and effect modifiers. Give diagnostic criteria, if applicable	6-7
Data sources/ measurement	8*	For each variable of interest, give sources of data and details of methods of assessment (measurement). Describe comparability of assessment methods if there is more than one group	*6-7*
Bias	9	Describe any efforts to address potential sources of bias	9
Study size	10	Explain how the study size was arrived at	5
Quantitative variables	11	Explain how quantitative variables were handled in the analyses. If applicable, describe which groupings were chosen and why	6-7, 8-9
Statistical methods	12	(*a*) Describe all statistical methods, including those used to control for confounding	8-9
		(*b*) Describe any methods used to examine subgroups and interactions	8-9
		(*c*) Explain how missing data were addressed	6-7,9
		(*d*) If applicable, explain how loss to follow-up was addressed	9
		(*e*) Describe any sensitivity analyses	9

For peer review only

**Results**			
Participants	13*	(a) Report numbers of individuals at each stage of study—eg numbers potentially eligible, examined for eligibility, confirmed eligible, included in the study, completing follow-up, and analysed	5-7, all tables
		(b) Give reasons for non-participation at each stage	5,16
		(c) Consider use of a flow diagram	-
Descriptive data	14*	(a) Give characteristics of study participants (eg demographic, clinical, social) and information on exposures and potential confounders	9, Table 1
		(b) Indicate number of participants with missing data for each variable of interest	Table 1
		(c) Summarise follow-up time (eg, average and total amount)	6
Outcome data	15*	Report numbers of outcome events or summary measures over time	6-12, all tables
Main results	16	(*a*) Give unadjusted estimates and, if applicable, confounder-adjusted estimates and their precision (eg, 95% confidence interval). Make clear which confounders were adjusted for and why they were included	9-12, all tables
		(*b*) Report category boundaries when continuous variables were categorized	All relevant tables
		(*c*) If relevant, consider translating estimates of relative risk into absolute risk for a meaningful time period	-
Other analyses	17	Report other analyses done—eg analyses of subgroups and interactions, and sensitivity analyses	12, all tables
**Discussion**			
Key results	18	Summarise key results with reference to study objectives	12-13
**Limitations**			
Interpretation	20	Give a cautious overall interpretation of results considering objectives, limitations, multiplicity of analyses, results from similar studies, and other relevant evidence	14-15
Generalisability	21	Discuss the generalisability (external validity) of the study results	15-16
**Other information**			
Funding	22	Give the source of funding and the role of the funders for the present study and, if applicable, for the original study on which the present article is based	24

*Give information separately for cases and controls in case-control studies and, if applicable, for exposed and unexposed groups in cohort and cross-sectional studies.

Note: An Explanation and Elaboration article discusses each checklist item and gives methodological background and published examples of transparent reporting. The STROBE checklist is best used in conjunction with this article (freely available on the Web sites of PLoS Medicine at http://www.plosmedicine.org/, Annals of Internal Medicine at http://www.annals.org/, and Epidemiology at http://www.epidem.com/). Information on the STROBE Initiative is available at www.strobe-statement.org.

For peer review only

BMJ Open

Developmental factors associated with decline in grip strength from midlife to old age: a British birth cohort study

Journal:	*BMJ Open*
Manuscript ID	bmjopen-2018-025755.R1
Article Type:	Research
Date Submitted by the Author:	05-Dec-2018
Complete List of Authors:	Kuh, Diana; MRC Unit for Lifelong Health and Ageing at UCL,<br>Hardy, Rebecca; University College London<br>Blodgett, Joanna; University College London<br>Cooper, Rachel; UCL, MRC Unit for Lifelong Health and Ageing at UCL
**Primary Subject Heading**:	Epidemiology
Secondary Subject Heading:	Public health
Keywords:	muscle strength, ageing, life course, development, birth cohort

SCHOLARONE™
Manuscripts

Developmental factors associated with decline in grip strength from midlife to old age: a British birth cohort study

Kuh D[1], Hardy R, Blodgett JM[1], Cooper R[1]

[1]MRC Unit for Lifelong Health and Ageing at UCL, London

Abbreviated title: Lifetime risk factors on grip strength decline

Word count: 3997

Corresponding author

Professor Diana Kuh PhD FMedSci

MRC Unit for Lifelong Health and Ageing at UCL

33 Bedford Place

London

WC1B 5JU

Tel: + 44 (0) 20 7670 5701

e-mail: d.kuh@ucl.ac.uk

For peer review only

Abstract

Objectives: To test whether developmental factors are associated with grip strength trajectories between 53 and 69 years, and operate independently or on the same pathway/s as adult factors.

Design: British birth cohort study

Setting: England, Scotland and Wales

Participants: 3058 men and women

Main outcome measures: Grip strength (kg) at ages 53, 60-64 and 69, were analysed using multilevel models to estimate associations with developmental factors (birthweight, growth parameters, motor and cognitive development) and father's social class, and investigate adult factors that could explain observed associations, testing for age and sex interactions.

Results: In men, heavier birthweight, beginning to walk 'on time', later puberty and greater weight 0-26 years and in women, heavier birthweight and earlier age at first standing in women, were independently associated with stronger grip but not with its decline. The slower decline in grip strength (by 0.07 kg/year, 95% confidence interval (CI) 0.02, 0.11 per 1SD, p=.003) in men of higher cognitive ability was attenuated by adjusting for adult verbal memory.

Conclusions: Patterns of growth and motor development have persisting associations with grip strength between midlife and old age. The strengthening associations with cognition suggest that, at older ages, grip strength increasingly reflects neural ageing processes. Interventions across life that promote muscle development or maintain muscle strength should increase the chance of an independent old age.

For peer review only

Article Summary

<u>Strengths and limitations of this study</u>

- This British birth cohort study has prospectively assessed factors from development onwards, has a wide range of potential covariates, and remains broadly representative of the population born in Britain in the early post war period
- There are repeat measures of grip strength assessed over a relatively long follow-up from midlife (53 years) to early old age (69 years), where there have been few studies of age-related decline.
- It was only possible to model linear change as there are currently only three assessments of grip strength; but we did investigate whether each association strengthened or weakened with age.
- While accounting for deaths and attrition, and those unable to provide a grip measurement for health reasons, did not alter our findings, the observed associations could still be explained by residual confounding.

Key words: muscle strength, ageing, life course trajectory, development, birth cohort

For peer review only

INTRODUCTION

Maintaining musculoskeletal function for the maximal period of time, and preventing musculoskeletal disorders are important aspects of healthy ageing, enabling people to remain active and independent for longer.[1,2] Worldwide, musculoskeletal disorders rank second in terms of impact on years lived with disability.[2] The role of muscle mass, strength and metabolic function is recognised for these disorders, and is becoming more widely appreciated in cardiovascular and other chronic diseases.[3,4]

Hand grip strength is a commonly used indicator of muscle strength;[5] and an overall biomarker of ageing.[6-8] Average levels rise to a peak during the early 30s, plateau and then decline.[9-11] Weaker grip is associated with future morbidity, disability and mortality across populations of different ages, ethnicities and income levels,[4,12-18] as is decline in grip strength.[19,20] Adult risk factors, including height and adiposity, health conditions, cognition and health behaviours, have been associated with subsequent grip strength,[21,22] and with age-related decline.[23-34] Developmental factors, such as birthweight, physical growth, motor and cognitive development, and childhood socioeconomic conditions are also related to adult grip strength[35-37] but evidence on whether they are associated with age-related decline is limited.[34,38,39] Using two repeat measures of midlife grip strength in the Medical Research Council (MRC) National Survey of Health and Development (NSHD, the oldest of the British birth cohort studies), Cooper et al. showed that those of higher childhood cognitive ability and socioeconomic position (SEP) (but not higher birthweight) had a reduced risk of decline in grip strength between 53 and 60-64 years.[34] Findings from the Lothian Birth cohort 1921 showed that during late life (79-87 years) those from a lower childhood SEP had a greater decline in grip strength,[39] but there was no association with childhood cognitive ability.[38]

For peer review only

A research gap is to understand whether developmental factors operate on the rate of decline in grip strength independently or on the same pathway/s as adult factors to inform the timing and types of interventions that may modify this decline. Based on previous findings,[34,36,39] and using three repeat measures of grip strength ascertained from age 53 to 69 years in the NSHD, we tested two hypotheses: (1) higher birthweight, greater gains in height and weight during childhood and adolescence, and earlier puberty are associated with a greater grip strength but not its decline; and (2) higher childhood cognitive ability, achieving motor milestones early or around the modal age, and higher childhood SEP, are associated with greater grip strength and a slower rate of decline. Further, we expected that any associations between indicators of physical growth and grip strength to be explained by adult health conditions and body mass index (BMI), and that any associations between motor and cognitive development, childhood SEP and grip strength to be explained by education and adult cognition.

METHODS

Sample

The Medical Research Council National Survey of Health and Development (NSHD) is a sample of all births in one week in March 1946 in mainland Britain comprising 5,362 (2,547 female) individuals followed up 24 times, so far to age 69.[40] The maximum sample for these analyses comprised 3,058 participants with at least one measure of grip strength at ages 53, 60-64 or 69 years and known adult height and birthweight. Of the remaining 2,304 in the original birth cohort, 738 had died, 542 were living abroad, 270 had been lost to follow-up, and 166 had not provided all necessary data. Those still participating in the NSHD in adulthood have lower childhood SEP, and higher adult SEP and childhood cognitive ability than those who did not, but the sample remains generally representative of those born in

For peer review only

Britain in the mid-twentieth century.[41,42] Ethical approval for the most recent visit was given by Queen Square Research Ethics Committee (13/LO/1073) and Scotland A Research Ethics Committee (14/SS/1009). Participants provided written informed consent for each visit.

Grip strength

During nurse assessments at ages 53 and 60-64, grip strength was measured in kilograms isometrically using a Nottingham electronic handgrip dynamometer; during a nurse home visit at age 69, a Jamar Plus+ Digital Hand dynamometer was used. A randomised repeated-measurements cross-over trial found no statistically significant differences in values when comparing these devices.[43] We applied the same standardised protocols and used the maximum of the first four measures (two in each hand) at each age.

Childhood factors

Birthweight: Birthweight, extracted from birth records to the nearest quarter pound, was converted to kilograms.

Physical growth: The SITAR model of growth curve analysis was used to estimate individual patterns of growth in height and weight between 0 and 26 years.[44,45] Heights and weights were measured using standardised protocols at ages 2, 4, 6, 7, 11 and 15, and self-reported at ages 20 and 26. The NSHD data were augmented by height and weight data between 5 and 19 years from the ALSPAC cohort. Subject-specific random effects were obtained for size, tempo and velocity (in SD units) for height and weight.[44] Later puberty is indicated by positive tempo values and earlier puberty by negative values.[44]

Motor and cognitive development: Age (in months) at first sitting, standing and walking was based on maternal recall at age 2. At age 15, a standardised measure of childhood cognitive ability was derived from the Heim AH4 test of fluid intelligence, the Watts Vernon reading

For peer review only - http://bmjopen.bmj.com/site/about/guidelines.xhtml

test and a test of mathematical ability.[46,47] Standardised scores (mean=0; SD =1) from similar tests at ages 11 or 8 were used if missing at age 15 as participants maintained similar ranking across time; Pearson correlation coefficients between the overall cognitive test scores at these three ages were between 0.7 and 0.9.[34]

Childhood SEP: Father's occupation at age 4 (or at age 11 or age 15 if missing at age 4), based on the Registrar General's Social Classification, distinguished three groups: high (I or II), middle (IINM or IIM) and low (IV and V).

Adult factors

Height and adiposity: At ages 53, 60-64 and 69, height (cm) and weight (kg) were measured using standard protocols and BMI (kg/m^2) was calculated; standardised scores were used in analyses.

Health conditions: At age 53, a summary of health conditions was a count (0-4) of the presence of knee osteoarthritis, hand osteoarthritis (both based on clinical assessment), severe respiratory symptoms and other potentially disabling or life threatening conditions.[25]

Education and verbal memory: Highest educational attainment by age 26 distinguished those with a degree or higher, advanced secondary, ordinary secondary, other or no qualifications. At ages 53, 60-64 and 69, verbal memory was assessed using a 15 item word list task over three trials (range 0-45),[48] and was converted to a standardised score with a mean of 0 and a standard deviation (SD) of 1.

Other adult covariates: At ages 53, 60-64 and 69, participants reported if they smoked and how many times they had taken part in any sports or vigorous leisure activities in the last month (grouped into more than 5 times a month, 1-4 times a month or not at all). Adult SEP, assessed by own occupation at age 53 (or at earlier ages if missing at age 53), distinguished the same three groups as for father's occupation.

For peer review only

Statistical analysis

Stata v14.2 was used for all analyses. We fitted multilevel models which account for the correlation of repeated measures of grip strength within individuals with age centred at 53 years. Preliminary multilevel models tested whether adult height was associated with grip strength and remained constant with age,[33] and whether men had stronger grip but a faster rate of decline than women, as expected.[23,28,29,33,38,49,50]

In the main analysis, models were run separately for men and women because of evidence of sex interactions with age and other covariates; these are reported where p <0.1. All models were adjusted for height, with change in grip strength modelled by a linear age term, and with the intercept and slope fitted as random effects. We also assessed whether associations changed with age (i.e. whether the risk factor was associated with slope) by testing for interactions between each risk factor and age. We tested for non-linearity with the continuous risk factors (by including a quadratic term). For parsimony, only those age or quadratic terms where p<0.1 remained in the models and are presented in the tables.

To investigate developmental risk factors and grip strength, we first investigated separately the associations with birthweight, physical growth, motor development, cognitive development and childhood SEP. For physical growth, we included all SITAR parameters in the same multilevel models. For motor development, we ran models for age at first sitting, standing and walking, first separately and then mutually adjusted.

Then we took the developmental factors associated with grip strength (p<0.05) and adjusted in turn for each group of adult factors, having shown in supplementary analyses how these

For peer review only

adult factors were associated with grip strength. Further details of the models are provided in Supplementary material.

In sensitivity analyses, to assess potential bias introduced by: (a) excluding those participants with no valid observations who were unable to perform the test for health reasons, and (b) mortality or other attrition during follow-up, we reran the multilevel models (1) giving a value representing the midpoint of the lowest sex-specific fifth of grip strength to participants unable for health reasons (n=29, 81 observations); and (2) including binary indicators for mortality (n=287) and attrition (n=601).[27,51]

Participant and patient involvement

Over the 70 years of the study, the research team has increasingly involved participants, in line with changing norms about conducting cohort studies. Findings from this analysis will be reported with the annual dissemination of study findings in birthday cards that participants have received since age 16, and lay summaries will be placed on the website. Participant involvement includes receiving personal letters from the research team as required, and invitations to participate in birthday celebrations, public engagement activities, and focus groups to discuss future data collections. When piloting new questionnaires and assessments, we recruit patients from GP practices or from the UCLH Patient Public Involvement (PPI) group, and take into account their feedback when designing the mainstage fieldwork.

RESULTS

Characteristics of the sample and preliminary analyses

Mean levels of grip strength, birthweight, and adult height were greater in men than women; women had more health conditions and lower SEP than men (Table 1). Mean grip strength

For peer review only

declined between ages 53 and 69 by 7.5kg for men and 3.6kg for women; thus the difference between men and women attenuated over time, although the mean sex difference remained substantial at age 69 (16.1kg) (Table 1). Preliminary multilevel models confirmed that adult height was strongly associated with grip strength (3.2 kg, 95% confidence interval (CI) 2.8,3.5 per 1SD increase in height, p=<.001) and remained constant with age, and that men had stronger grip and a faster rate of decline than women (p-value<.001 for sex interaction with age).

<u>Developmental factors: multilevel models</u>

Birthweight and physical growth

In models adjusted for adult height and age, there were positive associations between birthweight and grip strength which remained constant at all ages for men (p-value =0.7 for the birthweight by age interaction) but became weaker with age for women (p-value=0.05 for the interaction) (Table 2a). The association was stronger in men. In men, having a greater weight between birth and 26 years, and a later puberty (as indicated by a positive coefficient for height tempo) was associated with stronger grip. In women, shorter height, greater weight and a slower weight velocity between birth and 26 years were associated with stronger grip (Table 2b). These associations did not change with age.

Motor development

Later ages of attaining infant motor milestones of first sitting and standing were associated with weaker grip; there was an inverse U-shaped relationship between age at first walking and grip strength in men (Table 2c-e). These associations remained constant with age as there was no evidence of age interactions. When all three motor milestones, together with height were included together, only the inverse U-shaped relationship between age at first walking

For peer review only

and grip strength (for men) and the inverse association between age at first standing and grip strength (for women) remained (Supplementary Table 1); so these variables were carried forward.

Cognitive development

In men, there was an inverse U-shaped relationship between childhood cognitive ability and grip strength at age 53, as indicated by the negative estimates for cognition and cognition squared (Table 2f). The positive age interactions with both linear and quadratic cognition terms indicate that the association of childhood cognition with grip strength changes with age. Plotting the estimated mean grip strength for this model for the mean cognitive score, and ± 1SD showed that men of higher childhood cognition had lower grip strength than those with a mean cognitive score at age 53 but a slower decline in grip strength (by 0.07 kg/year, 95% confidence interval (CI) 0.02, 0.11 per 1SD, P=0.003) and thus a higher grip strength at age 69 (Figure 1). In women, higher childhood cognitive ability was associated with stronger grip and this remained constant with age.

Childhood SEP

There was no association between childhood SEP and grip strength in men (Table 2g). However, in women there was weak evidence that the association grew stronger with age; women from social classes IV and V showed a faster decline in grip strength (Figure 2).

Mutual adjustment of childhood factors

Mutual adjustment of birthweight, physical growth, age at walking (men), age at standing (women), and cognitive development and childhood SEP identified the factors most associated with stronger grip. In men, these were higher birthweight and childhood weight, later puberty, the non-linear relationship with age at first walking and the non-linear relationship with childhood cognition which became increasingly linear with age

For peer review only

(Supplementary Table 2). In women, these were higher birthweight and higher childhood cognition; the estimates for age at first standing were somewhat reduced in this sample with complete childhood data (Supplementary Table 3).

Adult factors: multilevel models

All adult covariates were associated with adult grip strength (supplementary Table 4a-g). Of particular relevance for our hypotheses, higher BMI was associated with stronger grip for men (but not women) and this association levelled off at higher levels of BMI and became weaker with age. Having more health conditions was associated with lower grip strength; for men, this association strengthened with age but for women it weakened. Higher educational levels were associated with stronger grip, especially among women. The association between verbal memory and grip strength was slightly negative in men and not evident in women at age 53 but grew stronger and positive with age.

Developmental factors adjusted for adult covariates: multilevel models

In men, the estimates for birthweight, height tempo and weight 0-26 years changed little after adjusting in turn for each adult factor with the greatest reduction in the estimate for birthweight occurring when health conditions were included in the model (Table 3). In contrast, the increasing association between childhood cognitive ability and grip strength with age was strongly attenuated by including verbal memory and a verbal memory by age interaction in the model (Table 4); other adult risk factors had much less impact.

In women, the association between childhood cognitive ability and grip strength was reduced by several adult factors, but especially by educational level (Table 5). However, the association between birthweight and grip strength was not reduced by any of the adult factors.

For peer review only

The estimates for age at first walking (in men) and age at first standing (in women) were lower in the sample with complete data; however, these lower estimates were not affected by adjustment for adult factors (Supplementary Table 5).

Sensitivity analyses

Giving a value for grip strength representing the mid-point of the lowest sex specific fifth for those who had no measure because of health reasons (maximum of 81 observations) had either no change or marginal change on the estimates. Compared with participants who were followed up and assessed at age 69, those who died during follow-up had lower mean grip strength at age 53 (-2.4kg, 95% CI -4.4, -0.41 for men, P=0.02; and -2.5kg, 95% CI -3.9, -1.2 for women, P<0.001) and, for men, there was weak evidence of a faster decline in grip strength (by -0.23kg/year, 95% CI -0.37,0.014, P=0.06). The mean differences in grip strength between those dropping out for reasons other than death and those completing follow-up were smaller (-0.99kg, 95%CI -2.4,0.41 for men, P=0.2; and -1.0kg, 95% CI -1.9, -0.16 for women, P=0.02) with no evidence of age interaction. However, estimates for the associations between developmental and adult risk factors and grip strength remained similar after adjusting for death and attrition.

DISCUSSION

In a prospective, nationally representative British birth cohort, developmental indicators had persisting associations with grip strength over 16 years from midlife to old age. Physical growth, in terms of heavier birthweight and, for men, later puberty and greater weight throughout the growth period was associated with stronger grip, and these effects were robust to adjustment for adult factors. 'On time' motor development for males and advanced motor development for females were associated with stronger grip which were unexplained by adult

For peer review only

factors. Childhood cognitive ability was associated with stronger grip (women) and a slower decline in grip strength (men) during that same life stage; these associations were explained by later education in women and adult cognition in men.

Comparison with other studies

Comparisons between studies are difficult because different methods have been used to assess decline in grip strength, not all analyses adjust for current adult height, and few studies have developmental data. Some studies have only two grip assessments,[25,30,33,34,52] or take the difference between the average of several later assessments from the average of several earlier assessments:[32] both methods are limited in their ability to analyse risk factors related to change. Studies with three assessments used bivariate growth curve models[38] or multilevel models.[27] Other studies with five or more assessments used latent growth curve modelling.[28,53]

In NSHD there was no association between childhood SEP and adult grip strength or its decline after adjusting for developmental factors. Findings from other studies have not been consistent and have adjusted for few, if any, other childhood factors, or have not studied change.[34,36,39,53] The results of a meta-analysis showed modest associations between childhood SEP and adult grip strength at a single time point which were attenuated by adult SEP and current body size, but there was considerable heterogeneity between studies.[54]

The constant effect of birthweight on adult grip strength is consistent with a meta-analysis;[55] this showed a larger estimate for men than women (as this study found) but the sex interaction was not significant. The persisting associations between growth parameters, motor

For peer review only

milestones and grip strength are novel findings, and build on previous NSHD work relating to grip strength at age 53,[36] and bone phenotype at age 60-64.[45,56]

The most striking observation in this study was the strengthening of the positive associations between cognition and grip strength with age, whether cognition was assessed in childhood or adult life. This extends an earlier NSHD study showing that the group with meaningful decline in grip strength between ages 53 and 60-64 had lower childhood cognitive ability than those who experienced no meaningful change.[34] In older cohorts, there is growing evidence that changes in grip strength are related to baseline cognition, and that cognitive decline may precede declines in strength,[24] although the few studies investigating covariation in cognition and grip strength have been inconsistent.[38,57] Our findings complement the findings from older cohorts as they cover midlife changes in grip strength over a longer follow-up period than most previous studies.

Explanation of Findings

Birthweight, physical growth and motor development

The persistence of the associations between birthweight, growth parameters, motor milestones and grip strength are worth noting given that more proximal factors may come into play as people age which could have diminished this association; however, the size of the estimates suggest that the associations may not be clinically meaningful.

Later puberty (in men) was associated with stronger grip, yet earlier puberty was associated with greater areal and volumetric bone mineral density in this cohort,[45] perhaps due to the differential impact of hormonal regulation. Nevertheless, we found that, controlling for contemporaneous body size, greater weight and slower weight velocity throughout the growth period was associated with both greater grip strength and greater bone size,[44] suggesting an extended growth period may benefit both. This could also be the explanation for the

For peer review only

persisting associations between motor milestones and grip strength and, in women, for the inverse association between height during growth and later grip strength, after controlling for adult height.

While the associations between physical growth and grip strength were independent of adult covariates, the number of health conditions attenuated the birthweight effect in men more than other covariates. Lower birthweight is predictive of CVD and diabetes,[58] as is poor muscle strength,[16] and may reflect a common pathway to later disease.

Lifetime cognition

Our findings regarding lifetime cognition and grip strength suggest that neural processes have greater impact on grip strength at older ages than in midlife. The attenuation of the childhood cognitive associations once verbal memory (for men) or education (for women) were taken into account may mean that neurodevelopmental processes play a role in maximising muscle function at maturity but neurodegenerative processes increasingly drive the age-related decline in muscle function. A theoretical model arising out of a review of cognitive ageing, motor learning and motor skills[59] predicts that ageing impairs cognitive functions before affecting the motor systems and that at older ages the connection between cognition and action becomes stronger, as suggested by our findings. To what extent our findings reflect a direct pathway between brain ageing and muscle strength,[60] or shared mechanisms relating, for example, to haemostatic dysregulation or inflammatory processes,[24,61] is yet to be clarified.

Strengths and Limitations

NSHD is one of the very few studies with prospectively assessed factors from development onwards, a wide range of potential covariates, and repeat measures of grip strength assessed

For peer review only

over a relatively long follow-up period during a critical phase of age-related change. So far these repeat measures cover midlife to early old age, a period which has been studied less often than later ages. NSHD remains broadly representative of the population born in Britain in the early post war period.[42] One limitation is that it is only possible to model linear change as there are currently only three assessments of grip strength. However we did investigate whether each association strengthened or weakened with age. Inevitably there were missing data but neither accounting for deaths and attrition, nor including those unable for health reasons, altered our findings. There were also few differences in the size of the estimates in the models using the maximum samples and the models with complete covariate data. We acknowledge that associations that were maintained after multiple adjustments could be due to residual confounding.

Implications

Muscle strength in later life is dependent on peak muscle function attained by young adulthood, and its subsequent rate of loss. Our findings that developmental as well as adult factors are associated with grip strength from midlife into old age suggest that primary prevention should start early in life and continue across life. Primary prevention should be supported by further research into the determinants of peak muscle function in order to develop the most effective strategies to maximise and maintain function. In a similar vein, a US report suggested it was time to shift the focus on to the primary prevention of osteoporosis, by better understanding the determinants of peak bone mass.[62] Given the associations between lifetime cognition and the level and change in muscle strength, trials to improve childhood or adult cognition should include muscle strength as an additional outcome.

For peer review only

CONCLUSIONS

Patterns of early growth, attainment of motor milestones, and lifetime cognition have persisting associations with grip strength between midlife and old age, even after taking account of adult body size, health conditions and health behaviours. The impact of neural processes strengthened over this stage of life suggesting that at older ages grip strength increasingly reflects both physical and cognitive ageing processes. Interventions across life that promote muscle development or maintain peak muscle strength should increase the chance of an active and independent old age.

For peer review only

Table 1. Characteristics of the Sample of 1,528 Men and 1,530 Women in the MRC National Survey of Health and Development With at Least One Measure of Grip Strength at ages 53, 60-64, or 69 and known Height and Birthweight

	Men		Women	
	N	Mean (SD) or %	N	Mean (SD) or %
Grip strength (kg)				
53y	1,398	47.7 (12.2)	1,434	27.7 (7.9)
60-64y	1,003	44.6 (11.6)	1,059	26.0 (7.4)
69y	1,036	40.2 (8.5)	1,062	24.1 (5.8)
Physical growth				
Birthweight (kg)	1,528	3.5 (0.5)	1,530	3.3 (0.5)
Height growth parameters 2-26y	1,509		1,505	
height (cm)		0.095 (6.1)		0.014 (5.8)
height-tempo (%)		-0.079 (6.1)		-0.19 (7.2)
height-velocity (%)		0.23 (10.0)		0.12 (10.6)
Weight growth parameters 0-26y	1,509		1,505	
weight (kg)		-0.25 (4.3)		-0.47 (4.3.)
weight tempo (%)		-0.49 (9.8)		-0.20 (7.7)
weight velocity (%)		-1.6 (26.2)		-0.41 (28.2)
Motor development (months)				
Age at first sitting	1,414	6.6 (1.5)	1,424	6.6 (1.5)
Age at first standing	1,416	11.4 (2.3)	1,419	11.3 (2.1)
Age at first walking	1,424	13.6 (2.5)	1,416	13.6 (2.4)
Early socioeconomic conditions				
Father's occupational class				
I & II	341	23.5	339	23.5
III	707	48.7	719	49.8
IV & V	405	27.9	385	26.7
ADULT FACTORS				
Height (cm)				
53y	1,428	174.7 (6.6)	1,477	161.6 (5.9)
60-64y	1,060	174.8 (6.6)	1,148	161.6 (5.9)
69y	1,038	173.9 (6.4)	1,079	160.6 (6.0)
BMI (kg/m^2)				
53y	1,427	27.4 (4.)	1,466	27.4 (5.4)
63y	1,059	27.9 (4.1)	1,147	27.9 (5.5)
69y	1,038	28.2 (4.6)	1,075	28.2 (5.8)
Verbal memory (no. words)				
53y	1,386	23.0 (6.2)	1,450	24.9 (6.2)
63y	1,020	23.0 (5.9)	1,114	25.4 (6.0)
69y	1,010	21.1 (6.0)	1,057	23.1 (6.0)
No. health conditions 53y				
0	802	56.4	702	47.6

For peer review only

1	448	31.7	543	37.0
2	147	10.5	159	11.3
3+	18	1.4	57	4.1
Smoking status 53y				
Non or ex-smoker	1,094	76.5	1,150	77.7
Smoker	336	23.5	330	22.3
Smoking status 60-64y				
Non or ex-smoker	975	87.2	1,052	88.3
Smoker	136	12.2	140	11.7
Smoking status 69y				
Non or ex-smoker	1,065	89.7	1,140	91.3
Smoker	122	10.2	109	8.7
Leisure-time physical activity 53y				
Inactive	675	47.2	745	50.3
Intermediate	268	18.7	240	16.2
Active	486	34.0	495	33.4
Leisure-time physical activity 60-64y				
Inactive	679	65.2	745	62.9
Intermediate	136	13.0	240	14.4
Active	227	21.8	495	22.7
Leisure-time physical activity 69y				
Inactive	654	60.2	710	60.6
Intermediate	119	11.1	162	13.5
Active	314	28.9	256	25.9
Qualifications by 26y				
Degree or higher	211	14.6	80	5.5
'A-level' or equivalents	410	28.4	338	23.4
'O-level' or equivalents	209	14.5	371	25.7
Less than 'O-level'	90	6.2	133	9.2
None	525	36.3	522	36.1
Own occupational class 53y				
I & II	779	51.6	555	36.6
III	571	37.8	641	42.2
IV & V	161	10.7	322	21.2
Died during follow-up				
No	1,359	88.9	1,412	92.3
Yes	169	11.1	118	7.7
Other attrition during follow-up				
No	1,225	80.2	1,232	80.5
Yes	303	19.8	298	19.5

For peer review only

Standardised score for childhood cognitive ability not presented for 1,408 men and 1,410 women as mean=0 and SD=1

For peer review only

Table 2. Estimates From Multilevel Models Showing Mean Differences in Grip Strength (kg) and Mean Differences in Grip Strength Change (kg/year) for Each Childhood Factor in NSHD. All Models Adjusted for Age Term and Standardised Adult Height.

	MEN				WOMEN			
	N	Reg. Coeff.	95% CI	P-value+	N	Reg. Coeff.	95% CI	P-value+
PHYSICAL GROWTH								
a. Birthweight (kg)	1,528				1,530	0.51	-0.07,1.10	0.09
Birthweight		1.96	1.08,2.84	<0.001		1.07	0.25,1.88	0.01
Birthweight* age (year)		N/A				-0.06	-0.12,0.00	0.05
b. Growth parameters	1,509				1,505			
Height -size (cm)		-0.01	-0.22,0.20	0.9		-0.16	-0.29,-0.03	0.02
Height-tempo (%)		0.16	0.06,0.26	0.001		-0.02	-0.04,0.07	0.5
Height-velocity (%)		-0.02	-0.10,0.05	0.5		0.00	-0.04,0.04	0.9
Weight-size (kg)		0.71	0.24,1.18	0.003		0.41	0.57,0.77	0.02
Weight-tempo (%)		-0.06	-0.12,0.01	0.08		-0.01	-0.06,0.04	0.8
Weight-velocity (%)		-0.07	-0.14,0.00	0.06		-0.06	-0.11,0.00	0.04
MOTOR DEVELOPMENT (Months)								
c. Age at sitting	1,414	-0.25	-0.57,0.06	0.1	1,424	-0.18	-0.38,0.01	0.07
d. Age at standing	1,416	-0.27	-0.47,-0.07	0.008	1,419	-0.21	-0.34,-0.07	0.002
e. Age at walking	1,424				1,416			
Walking		1.36	0.00,2.73	0.05		-0.13	-0.25,-0.01	0.03
Walking^2		-0.06	-0.10,-0.01	0.01		N/A		
f. COGNITIVE DEVELOPMENT(SD)	1,408				1,410			
Cognition		-0.46	-1.10,0.17	0.1		0.45	0.14,0.76	0.005
Cognition^2		-0.81	-1.27,-0.34	0.001		N/A		
Cognition*age (year)		0.07	0.02,0.11	0.003		N/A		
Cognition^2*age (year)		0.03	0.00,0.06	0.08		N/A		
g. FATHER'S OCCUPATIONAL CLASS	1,453				1,443			
I & II		REF		0.2		REF		0.4
III		-0.51	-1.64,0.62			-0.24	-1.15,0.67	
IV & V		-1.29	-2.58,0.00			0.38	-0.66,1.42	

For peer review only

III*age (year)		N/A				-0.03	-0.10,0.04	0.07
IV & V*age (year)		N/A				-0.10	-0.18,-0.01	

+ P-values are given for the overall tests of associations. P-values for sex interactions: birthweight p=.004; childhood height after adjustment for adult height p-value .07; height tempo p=.006; age at first waking (quadratic term) p=.02

For peer review only

Table 3. Estimates From Multilevel Models Showing Mean Differences in Grip Strength (kg) by Birthweight, Height Tempo and Weight-Size in 1,295 NSHD Men (2,788 Observations), Adjusted for Age Term, Standardised Adult Height and all Growth Parameters, and Then Additionally Adjusted for Each Set of Adult Factors in Turn

	Birthweight (kg)			Height Tempo (%)			Weight-size (kg)		
	Reg. Coeff.	95% CI	P-value	Reg. Coeff.	95% CI	P-value	Reg. Coeff.	95% CI	P-value
Adjusted for age, all growth parameters and adult height	1.35	0.33,2.37	0.009	0.25	0.14,0.35	<0.001	0.63	0.10,1.16	0.02
Additional adjustments in turn									
BMI[a], BMI^2, BMI*age	1.37	0.35,2.38	0.008	0.25	0.14,0.35	<0.001	0.60	0.07,1.12	0.03
Health conditions 53y Health conditions*age	1.28	0.27,2.29	0.01	0.24	0.13,0.35	<0.001	0.60	0.07,1.12	0.03
Qualifications	1.42	0.40,2.43	0.006	0.25	0.14,0.36	<0.001	0.61	0.08,1.14	0.02
Verbal memory[a], Verbal memory*age	1.39	0.37,2.41	0.008	0.24	0.13,0.35	<0.001	0.62	0.09,1.15	0.02
Own social class 53y, own social class*age	1.42	0.40,2.43	0.006	0.24	0.14,0.35	<0.001	0.60	0.07,1.12	0.03
Smoking & physical activity[a]	1.39	0.38,2.40	0.007	0.24	0.13,0.34	<0.001	0.63	0.10,1.15	0.02

For peer review only

Table 4. Estimates From Multilevel Models Showing Mean Differences in Grip Strength (kg) and Mean Differences in Grip Strength Change (kg/year) by Childhood Cognition in 1,161 NSHD Men (2,515 Observations), Adjusted for Age Term, Standardised Adult height and Age at First Walking, and Then Additionally Adjusted for Each Set of Adult Factors in Turn

	Childhood cognition (SD)			Childhood cognition $(SD)^2$			Childhood cognition (SD)*age (year)		
	Reg. Coeff.	95% CI	P-value	Reg. Coeff.	95% CI	P-value	Reg. Coeff.	95% CI	P-value
Adjusted for age, adult height, walking, $walking^2$	-0.44	-1.14,0.25	0.2	-0.52	-0.94,-0.11	0.01	0.08	0.028,0.13	0.002
Additional adjustments in turn									
BMI[a], BMI^2, BMI*age	-0.37	-1.06,0.32	0.3	-0.51	-0.93,-0.10	0.01	0.07	0.02,0.12	0.005
Health conditions 53y Health conditions*age	-0.55	-1.25,0.15	0.1	-0.53	-0.95,-0.12	0.01	0.08	0.03,0.13	0.002
Qualifications	-0.79	-1.62,0.04	0.06	-0.56	-0.98,0.14	0.009	0.08	0.03,0.13	0.002
Verbal memory [a], verbal memory*age	-0.12	-0.93,0.69	0.7	-0.54	-0.96,-0.12	0.01	0.03	-0.03.0.09	0.3
Own social class 53y, own social class*age	-0.80	-1.59,0.00	0.05	-0.52	-0.94,-0.10	0.01	0.07	-0.02,0.13	0.01
Smoking & physical activity[a]	-0.64	-1.34,0.06	0.07	-0.56	-0.97,-0.14	0.08	0.08	0.03,0.13	0.001

[a]time varying covariates;

For peer review only

Table 5. Estimates From Multilevel Models Showing Mean Differences in Grip Strength (kg) by Birthweight and Childhood Cognition in 1,211 NSHD Women (2,709 Observations), Adjusted for Age Term and Standardised Adult Height, and Then Additionally Adjusted for Each Set of Adult Factors in Turn.

	Birthweight (kg)			Birthweight*age (year)			Childhood cognition (SD)		
	Reg. Coeff.	95% CI	P-value	Reg. Coeff.	95% CI	P-value	Reg. Coeff.	95% CI	P-value
Adjusted for age, adult height, age at first standing and mutually adjusted	1.25	0.34,2.16	0.007	-0.08	-0.15,-0.02	0.01	0.37	0.031,0.71	0.03
Additional adjustments in turn									
BMI[a]	1.26	0.35,2.17	0.007	-0.08	-0.15,-0.02	0.01	0.36	0.02,0.70	0.04
Health conditions 53y[a], health conditions*age	1.27	0.37,2.19	0.006	-0.09	-0.15,-0.02	0.01	0.27	-0.07,0.61	0.1
Qualifications[a]	1.22	0.31,2.13	0.008	-0.08	-0.15,-0.02	0.01	0.019	-0.4,0.48	0.9
Verbal memory[a], verbal memory*age	1.29	0.38,2.20	0.005	-0.09	-0.16,-0.02	0.008	0.22	-0.17,0.61	0.3
Own social class	1.23	0.32,2.13	0.008	-0.09	-0.15,-0.02	0.01	0.27	-0.10,0.65	0.1
Smoking & physical activity[a]	1.37	0.47,2.26	0.003	-0.09	-0.16,-0.02	0.009	0.30	-0.04,0.65	0.08

[a] time varying covariates;

For peer review only

ACKNOWLEDGEMENTS

We thank NSHD study members for their lifelong participation and past and present members of the NSHD study team who helped to collect the data.

FUNDING

This work was supported by the UK Medical Research Council MC_UU_12019/1 which provides core funding for the MRC National Survey of Health and Development and supports DK, JB, RC, and RH by MC_UU_12019/1, MC_UU_12019/2, MC_UU_12019/4. JB also receives support from UCL (Overseas and Graduate Research Scholarships). The funders had no role in the study or the decision to submit the paper for publication.

AUTHOR CONTRIBUTIONS

DK, RC, JB and RH contributed to the study design and data interpretation and DK, RC and RH collected the data. DK undertook the literature search, the data analysis and wrote the first draft of the manuscript; all authors revised the manuscript. DK is the guarantor and accepts full responsibility for the work and the conduct of the study, had access to the data, and controlled the decision to publish. All authors, external and internal, had full access to all of the data (including statistical reports and tables) in the study and can take responsibility for the integrity of the data and the accuracy of the data analysis.

OPEN ACCESS: This is an Open Access article distributed in accordance with the terms of the Creative Commons Attribution (CC BY 3.0) license, which permits others to distribute, remix, adapt and build upon this work, for commercial use, provided the original work is properly cited..

For peer review only

DECLARATION OF COMPETING INTERESTS: All authors have completed the ICMJE uniform disclosure form and declare: DK, JB, RC, and RH received financial support from the UK Medical Research Council for the submitted work. JB also receives support from UCL (Overseas and Graduate Research Scholarships); no financial relationships with any organisations that might have an interest in the submitted work in the previous three years; no other relationships or activities that could appear to have influenced the submitted work.

DATA SHARING STATEMENT: Data used in this publication are available to bona fide researchers upon request to the NSHD Data Sharing Committee via a standard application procedure. Further details can be found at http://www.nshd.mrc.ac.uk/data. doi:10.5522/NSHD/Q101; doi:10.5522/NSHD/Q102; 10.5522/NSHD/Q103

For peer review only

Figure Legends

Figure 1. Mean grip strength (kg) by childhood cognition for men of mean height (based on 1408 men)

Figure 2. Mean grip strength (kg) by father's social class for women of mean height (based on 1443 women)

For peer review only

References

1. Kuh D, Karunananthan S, Bergman H, Cooper R. A life-course approach to healthy ageing: maintaining physical capability. *Proc Nutr Soc* 2014; **73**(2): 237-48.
2. Briggs AM, Cross MJ, Hoy DG, et al. Musculoskeletal Health Conditions Represent a Global Threat to Healthy Aging: A Report for the 2015 World Health Organization World Report on Ageing and Health. *Gerontologist* 2016; 56 Suppl 2: S243-55.
3. Wolfe RR. The underappreciated role of muscle in health and disease. *Am J Clin Nutr* 2006; **84**(3): 475-82.
4. Manring H, Abreu E, Brotto L, Weisleder N, Brotto M. Novel excitation-contraction coupling related genes reveal aspects of muscle weakness beyond atrophy-new hopes for treatment of musculoskeletal diseases. *Frontiers in Physiol* 2014; **5**: 37.
5. Bohannon RW. Are hand-grip and knee extension strength reflective of a common construct? *Percept Mot Skills* 2012; **114**(2): 514-8.
6. Martin-Ruiz CM, von Zglinicki T. A life course approach to biomarkers of ageing. In: Kuh D, Cooper R, Hardy R, Richards M, Ben-Shlomo Y, eds. A life course approach to healthy ageing. 1st ed. Oxford: Oxford University Press; 2014: 177-86.
7. Sayer AA, Kirkwood TB. Grip strength and mortality: a biomarker of ageing? *Lancet* 2015; **386**(9990): 226-7.
8. Justice JN, Cesari M, Seals DR, Shively CA, Carter CS. Comparative Approaches to Understanding the Relation Between Aging and Physical Function. *J Gerontol A Biol Sci Med Sci* 2016; **71**(10): 1243-53.
9. Dodds RM, Syddall HE, Cooper R, et al. Grip strength across the life course: normative data from twelve British studies. *PLoS ONE* 2014; **9**(12): e113637.
10. Dodds RM, Syddall HE, Cooper R, Kuh D, Cooper C, Sayer AA. Global variation in grip strength: a systematic review and meta-analysis of normative data. *Age Ageing* 2016; **45**(2): 209-16.
11. Nahhas RW, Choh AC, Lee M, et al. Bayesian longitudinal plateau model of adult grip strength. *Am J Hum Biol* 2010; **22**(5): 648-56.
12. Rantanen T, Volpato S, Ferrucci L, Heikkinen E, Fried LP, Guralnik JM. Handgrip strength and cause-specific and total mortality in older disabled women: exploring the mechanism. *JAm Geriatr Soc* 2003; **51**(5): 636-41.

13. Cooper R, Kuh D, Hardy R. Objectively measured physical capability levels and mortality: systematic review and meta-analysis. *BMJ* 2010; **341**: c4467.
14. Cooper R, Kuh D, Cooper C, et al. Objective measures of physical capability and subsequent health: a systematic review. *Age Ageing* 2011; **40**: 14-23.
15. den Ouden ME, Schuurmans MJ, Arts IE, van der Schouw YT. Physical performance characteristics related to disability in older persons: a systematic review. *Maturitas* 2011; **69**(3): 208-19.
16. Leong DP, Teo KK, Rangarajan S, et al. Prognostic value of grip strength: findings from the Prospective Urban Rural Epidemiology (PURE) study. *Lancet* 2015; **386**(9990): 266-73.
17. Ortega FB, Silventoinen K, Tynelius P, Rasmussen F. Muscular strength in male adolescents and premature death: cohort study of one million participants. *BMJ* 2012; **345**: e7279.
18. Kim Y, Wijndaele K, Lee DC, Sharp SJ, Wareham N, Brage S. Independent and joint associations of grip strength and adiposity with all-cause and cardiovascular disease mortality in 403,199 adults: the UK Biobank study. *Am J Clin Nutr* 2017; **106**(3): 773-82.
19. Peterson MD, Zhang P, Duchowny KA, Markides KS, Ottenbacher KJ, Snih SA. Declines in Strength and Mortality Risk Among Older Mexican Americans: Joint Modeling of Survival and Longitudinal Data. *J Gerontol A Biol Sci Med Sci* 2016; **71**(12): 1646-52.
20. Syddall HE, Westbury LD, Dodds R, Dennison E, Cooper C, Sayer AA. Mortality in the Hertfordshire Ageing Study: association with level and loss of hand grip strength in later life. *Age Ageing* 2017; **46**(3): 407-12.
21. Shaw SC, Dennison EM, Cooper C. Epidemiology of Sarcopenia: Determinants Throughout the Lifecourse. *Calcif Tissue Int* 2017; **101**(3): 229-47.
22. Clouston SAP, Brewster P, Kuh D, et al. The dynamic relationship between physical function and cognition in longitudinal aging cohorts. *Epidemiologic Reviews* 2013; **35**(1): 33-50.
23. Syddall HE, Westbury LD, Shaw SC, Dennison EM, Cooper C, Gale CR. Correlates of Level and Loss of Grip Strength in Later Life: Findings from the English Longitudinal Study of Ageing and the Hertfordshire Cohort Study. *Calcif Tissue Int* 2017.
24. Fritz NE, McCarthy CJ, Adamo DE. Handgrip strength as a means of monitoring progression of cognitive decline - A scoping review. *Ageing ResRev* 2017; **35**: 112-23.
25. Cooper R, Muniz-Terrera G, Kuh D. Associations of behavioural risk factors and health status with changes in physical capability over 10 years of follow-up: the MRC National Survey of Health and Development. *BMJ Open* 2016; **6**(4): e009962.
26. Granic A, Davies K, Jagger C, Kirkwood TB, Syddall HE, Sayer AA. Grip Strength Decline and Its Determinants in the Very Old: Longitudinal Findings from the Newcastle 85+ Study. *PLoSOne* 2016; **11**(9): e0163183.
27. Botoseneanu A, Bennett JM, Nyquist L, et al. Cardiometabolic Risk, Socio-Psychological Factors, and Trajectory of Grip Strength Among Older Japanese Adults. *JAging Health* 2015; **27**(7): 1123-46.
28. Sternang O, Reynolds CA, Finkel D, Ernsth-Bravell M, Pedersen NL, Dahl Aslan AK. Factors associated with grip strength decline in older adults. *Age Ageing* 2015; **44**(2): 269-74.
29. Stenholm S, Tiainen K, Rantanen T, et al. Long-Term Determinants of Muscle Strength Decline: Prospective Evidence from the 22-Year Mini-Finland Follow-Up Survey. *J Am Geriatr Soc* 2012; **60**(1): 77-85.
30. Miller DK, Malmstrom TK, Miller JP, Andresen EM, Schootman M, Wolinsky FD. Predictors of change in grip strength over 3 years in the African American health project. *J Aging Health* 2010; **22**(2): 183-96.

For peer review only - http://bmjopen.bmj.com/site/about/guidelines.xhtml

31. Rantanen T, Penninx BW, Masaki K, Lintunen T, Foley D, Guralnik JM. Depressed mood and body mass index as predictors of muscle strength decline in old men. *J Am Geriatr Soc* 2000; **48**(6): 613-7.
32. Rantanen T, Masaki K, Foley D, Izmirlian G, White L, Guralnik JM. Grip strength changes over 27 yr in Japanese-American men. *J Applied Physiol* 1998; **85**(6): 2047-53.
33. Clement FJ. Longitudinal and cross-sectional assessments of age changes in physical strength as related to sex, social class, and mental ability. *J Gerontol* 1974; **29**: 423-9.
34. Cooper R, Richards M, Kuh D. Childhood Cognitive Ability and Age-Related Changes in Physical Capability From Midlife: Findings From a British Birth Cohort Study. 2017; **79**(7): 785-91.
35. Aihie Sayer A, Cooper C, Evans JR, et al. Are rates of ageing determined in utero? *Age Ageing* 1998; **27**: 579-83.
36. Kuh D, Hardy R, Butterworth S, et al. Developmental origins of midlife grip strength: Findings from a birth cohort study. *J Gerontol Series A-Biol Sci Med Scis* 2006; **61**(7): 702-6.
37. Cooper R, Hardy R, Sayers A, Kuh D. A life course approach to physical capability. In: Kuh D, Cooper R, Hardy R, Richards M, Ben-Shlomo Y, eds. A life course approach to healthy ageing. 1st ed. Oxford: Oxford University Press; 2014: 16-31.
38. Deary IJ, Johnson W, Gow AJ, et al. Losing one's grip: a bivariate growth curve model of grip strength and nonverbal reasoning from age 79 to 87 years in the Lothian Birth Cohort 1921. *J Gerontol B Psychol Sci Soc Sci* 2011; **66**(6): 699-707.
39. Starr JM, Deary IJ. Socio-economic position predicts grip strength and its decline between 79 and 87 years: the Lothian Birth Cohort 1921. *Age and Ageing* 2011; **40**(6): 749-52.
40. Kuh D, Wong A, Shah I, et al. The MRC National Survey of Health and Development reaches age 70: maintaining participation at older ages in a birth cohort study. *Eur J Epidemiol* 2016; **31**(11): 1135-47.
41. Wadsworth ME, Butterworth SL, Hardy RJ, et al. The life course prospective design: an example of benefits and problems associated with study longevity. *Soc Sci Med* 2003; **57**(11): 2193-205.
42. Stafford M, Black S, Shah I, et al. Using a birth cohort to study ageing: representativeness and response rates in the National Survey of Health and Development. *Eur J Ageing* 2013; **10**(2): 145-57.
43. Lessof C. Testing for differences in measurement devices: findings from a randomized trial to compare measures of physiological function and physical performance European Survey Research Association; 2017 19th July; Lisbon; 2017.
44. Cole TJ, Kuh D, Johnson W, et al. Using Super-Imposition by Translation And Rotation (SITAR) to relate pubertal growth to bone health in later life: the Medical Research Council (MRC) National Survey of Health and Development. *Int J Epidemiol* 2016; **45**(4): 1125-34.
45. Kuh D, Muthuri SG, Moore A, et al. Pubertal timing and bone phenotype in early old age: findings from a British birth cohort study. *Int J Epidemiol* 2016; **45**(4): 1113-24.
46. Heim AW. The AH4 group test of intelligence. Windsor: NFER-Nelson; 1970.
47. Pigeon DA. Details of the fifteen years tests. In: JWB D, JM R, HR S, eds. All our future. London: Davies; 1968: Appendix 1.
48. Davis D, Bendayan R, Muniz G, R H, M R, Kuh. Decline in search speed and verbal memory over 26 years of midlife in a British birth cohort. *Neuroepidemiol* 2017;**49** (3-4):121-8.
49. Oksuzyan A, Maier H, McGue M, Vaupel JW, Christensen K. Sex Differences in the Level and Rate of Change of Physical Function and Grip Strength in the Danish 1905-Cohort Study. *J Aging Health* 2010; **22**(5): 589-610.

For peer review only - http://bmjopen.bmj.com/site/about/guidelines.xhtml

50. Botoseneanu A, Allore HG, Mendes de Leon CF, Gahbauer EA, Gill TM. Sex Differences in Concomitant Trajectories of Self-Reported Disability and Measured Physical Capacity in Older Adults. *J Gerontol A Biol Sci Med Sci* 2016; **71**(8): 1056-62.
51. Botoseneanu A, Liang J. The effect of stability and change in health behaviors on trajectories of body mass index in older Americans: a 14-year longitudinal study. *J Gerontol A Biol Sci Med Sci* 2012; **67**(10): 1075-84.
52. Daly RM, Ahlborg HG, Ringsberg K, Gardsell P, Sernbo I, Karlsson MK. Association between changes in habitual physical activity and changes in bone density, muscle strength, and functional performance in elderly men and women. *J Am Geriatr Soc* 2008; **56**(12): 2252-60.
53. Kroger H, Fritzell J, Hoffmann R. The Association of Levels of and Decline in Grip Strength in Old Age with Trajectories of Life Course Occupational Position. *PLoSOne* 2016; **11**(5): e0155954.
54. Birnie K, Cooper R, Martin RM, et al. Childhood socioeconomic position and objectively measured physical capability levels in adulthood: A systematic review and meta-analysis. *PLoS ONE* 2011; **6**(1): e15564.
55. Dodds R, Denison HJ, Ntani G, et al. Birth weight and muscle strength: A systematic review and meta-analysis. *J Nutr Health Ageing* 2012; **16**(7): 609-15.
56. Kuh D, Wills AK, Shah I, et al. Growth from birth to adulthood and bone phenotype in early old age: A British birth cohort study. *J Bone Miner Res* 2014; **29**(1): 123-33.
57. Praetorius Bjork M, Johansson B, Hassing LB. I forgot when I lost my grip-strong associations between cognition and grip strength in level of performance and change across time in relation to impending death. *Neurobiol aging* 2016; **38**: 68-72.
58. Whincup PH, Kaye SJ, Owen CG, et al. Birth weight and risk of type 2 diabetes: A systematic review. *JAMA* 2008; **300**(24): 2886-97.
59. Ren J, Wu YD, Chan JS, Yan JH. Cognitive aging affects motor performance and learning. *Geriatr Gerontol Int* 2013; **13**(1): 19-27.
60. Bhanushali M, Conwit R, Metter RM, Ferrucci L. The role of the nervous system in muscle atrophy. In: Cruz-Jentoft AJ,Morley JE, editors. Sarcopenia. Chichester, UK: John Wiley & Sons; 2012.
61. Newman AB, Sanders JL, Kizer JR, et al. Trajectories of function and biomarkers with age: the CHS All Stars Study. *Int J Epidemiol* 2016; **45**(4): 1135-45.

62. Gordon CM, Zemel BS, Wren TAL, Leonard MB, Bachrach LK, Rauch F, Gilsanz V, Rosen CJ, Winer KK. The determinants of peak bone mass. Workshop summary. J Paediat 180:261-269.

For peer review only

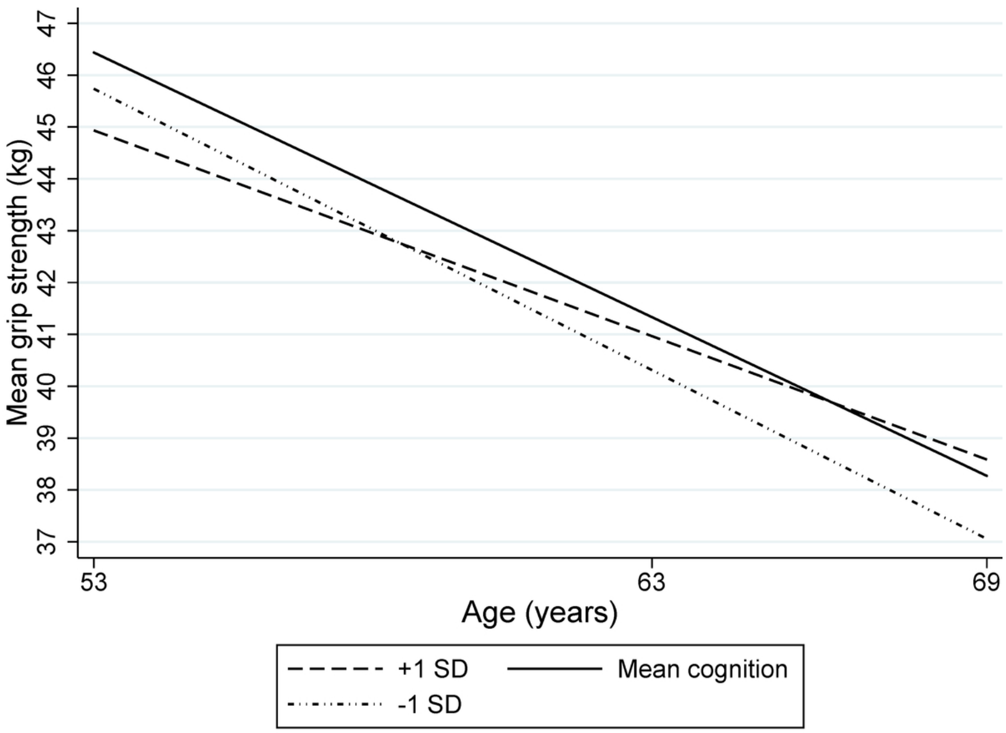

Figure 1. Mean grip strength (kg) by childhood cognition for men of mean height (based on 1408 men)

101x73mm (300 x 300 DPI)

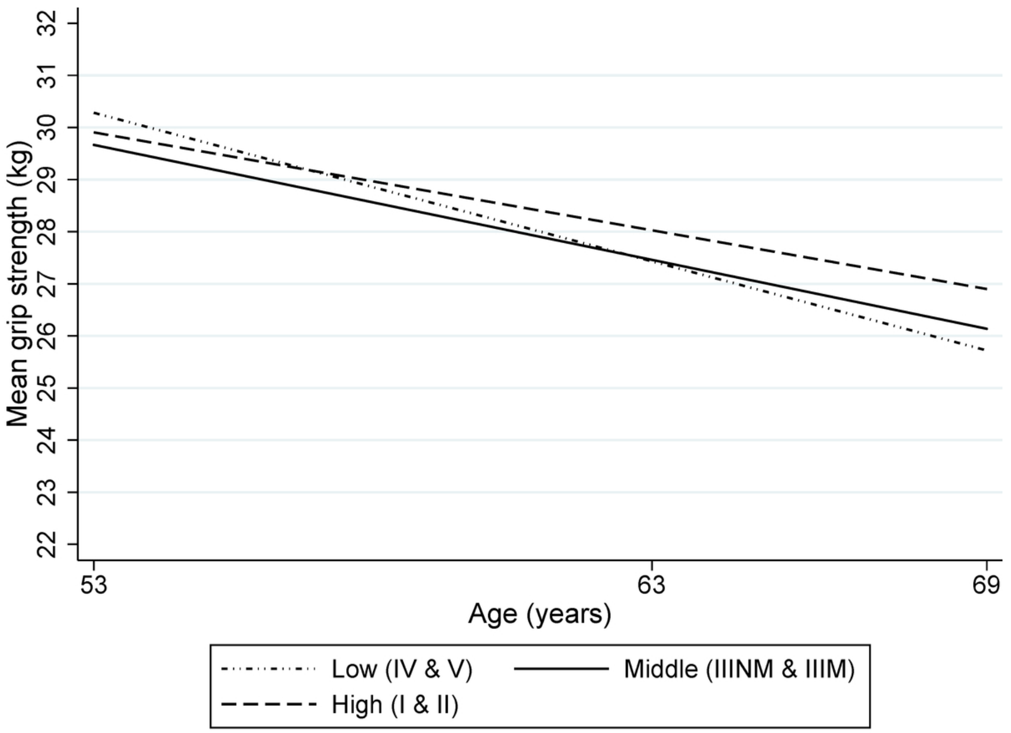

Figure 2. Mean grip strength (kg) by father's social class for women of mean height (based on 1443 women)

101x73mm (300 x 300 DPI)

Developmental factors associated with decline in grip strength from midlife to old age: a British birth cohort study

Kuh D[1], Hardy R, Blodgett JM[1], Cooper R[1]

[1]MRC Unit for Lifelong Health and Ageing at UCL, London

For peer review only

SUPPLEMENTARY MATERIAL

For peer review only - http://bmjopen.bmj.com/site/about/guidelines.xhtml

Supplementary Material

Model specification

Multilevel models were used to estimate the grip strength trajectory defined by the intercept (at age 53) and the slope, with measurements (level1) nested within individuals (level 2).

If $grip_{ij}$ denotes grip strength of individual *j (j=1,…,n)* on measurement occasion *i (i=1,2,3)* and age_{ij} the age (centred at 53 years) at which that measurement was recorded, the basic multilevel model can be written as:

$$grip_{ij} = \alpha + u_j + (\beta_1 + v_j)age_{ij} + e_{ij}$$

where the fixed parameter α is the mean intercept and the fixed parameter β the mean slope. The parameters u_j and v_j represent the deviation of the *j*th individual's intercept from the overall mean and slope, respectively. Finally, e_{ij} is the residual error term.

Covariates were then added to the model and could be associated with the intercept and the slope. Covariate by age interactions were assessed and included in models if p<0.1.

For Table 3 in men, the first line of the table is from the model:

$$grip_{ij} = \alpha + u_j + (\beta_1 + v_j)age_{ij} + \beta_2 bwt_j + \beta_3 htsize_j + \beta_4 httempo_j + \beta_5 htvelocity_j + \beta_6 wtsize_j + \beta_7 wttempo_j + \beta_8 wtvelocity_j + \beta_9 adultht_{ij} + e_{ij}$$

Adjusting for BMI, for example, as a time-varying covariate which is associated with both intercept (β_{10}, β_{11}) and slope (γ_1), the model is:

$$grip_{ij} = \alpha + u_j + (\beta_1 + v_j)age_{ij} + \beta_2 bwt_j + \beta_3 htsize_j + \beta_4 httempo_j + \beta_5 htvelocity_j + \beta_6 wtsize_j + \beta_7 wttempo_j + \beta_8 wtvelocity_j + \beta_9 adultht_{ij} + \beta_{10} BMI_{ij} + \beta_{11} BMI_{ij}^2 + \gamma_1 BMI_{ij}.age_{ij} + e_{ij}$$

Similarly, for Table 4 in men, the first line of the table where childhood cognition is associated with both intercept (β_2, β_3) and the slope (γ_1), the model is:

$$grip_{ij} = \alpha + u_j + (\beta_1 + v_j)age_{ij} + \beta_2 childcog_j + \beta_3 childcog_j^2 + \gamma_1 childcog_j.age_{ij} + \beta_4 walking_j + \beta_5 walking_j^2 + \gamma_2 walking_j.age_{ij} + \beta_6 adultht_{ij} + e_{ij}$$

Adjusting for adult BMI:

For peer review only - http://bmjopen.bmj.com/site/about/guidelines.xhtml

$$grip_{ij} = \alpha + u_j + (\beta_1 + v_j)age_{ij} + \beta_2 childcog_j + \beta_3 childcog_j^2 \\ + \gamma_1 childcog_j . age_{ij} + \beta_4 walking_j + \beta_5 walking_j^2 + \gamma_2 walking_j . age_{ij} \\ + \beta_6 adultht_{ij} + \beta_7 BMI_{ij} + \beta_8 BMI_{ij}^2 + \gamma_3 BMI_{ij} . age_{ij} + e_{ij}$$

For Table 5 in women, the first line is from the model:

$$grip_{ij} = \alpha + u_j + (\beta_1 + v_j)age_{ij} + \beta_2 bwt_j + \gamma_1 bwt_j . age_{ij} \\ + \beta_3 childcog_j + \beta_4 walking_j + \beta_5 adultht_{ij} + e_{ij}$$

Adjusting for adult BMI:

$$grip_{ij} = \alpha + u_j + (\beta_1 + v_j)age_{ij} + \beta_2 bwt_j + \gamma_1 bwt_j . age_{ij} \\ + \beta_3 childcog_j + \beta_4 walking_j + \beta_5 adultht_{ij} + \beta_6 BMI_{ij} + \beta_7 BMI_{ij}^2 \\ + \gamma_2 BMI_{ij} . age_{ij} + e_{ij}$$

For peer review only

For peer review only - http://bmjopen.bmj.com/site/about/guidelines.xhtml

Supplementary Table 1. Estimates From Multilevel Models Showing Mean Differences in Grip Strength (kg) in 1,375 NSHD Men (3,111 Observations) and in 1,385 NSHD Women (3,266 Observations) for Mutually Adjusted Physical Growth Indicators. All Models Adjusted for Age Term and Adult height and Using Sample With Complete Childhood Data.

	MEN			WOMEN		
	Reg. Coeff.	95% CI	P-value	Reg. Coeff.	95% CI	P-value
Age (months) at sitting	0.030	-0.35,0.40	0.9	-0.031	-0.27,0.21	0.8
Age (months) at standing	0.14	-0.23,0.46	0.6	-0.29	-0.53,-0.052	0.02
Age (months) at walking						
Walking	1.72	0.33,3.1	0.01	0.11	-0.093,0.32	0.3
$Walking^2$	-0.075	-0.12,-0.028	0.002	N/A		

Supplementary Table 2. Estimates From Multilevel Models Showing Mean Differences in Grip Strength (kg) and Mean Differences in Grip Strength Change (kg/year) in 1,316 NSHD Men (2,983 Observations) for Mutually Adjusted Childhood Factors. All Models Adjusted for Age Term and Standardised Adult Height.

	Adjusted for age term and height			Fully adjusted		
	Reg. Coeff.	95% CI	P-value+	Reg. Coeff.	95% CI	P-value+
BIRTHWEIGHT (kg)	2.00	1.05,2.95	<0.001	1.40	0.41,2.38	0.005
GROWTH PARAMETERS						
Height -size (cm)	-0.09	-0.32,0.14	0.4	-0.07	-0.30,0.16	0.5
Height-tempo (%)	0.16	0.052,0,26	0.004	0.15	0.046,0.26	0.005
Height-velocity (%)	-0.02	-0.10,0.06	0.6	-0.03	-0.10,0.05	0.5
Weight-size (kg)	0.93	0.41,1.44	<0.001	0.62	0.094,1.15	0.02
Weight-tempo (%)	-0.07	-0.13,0.00	0.051	-0.05	-0.12,0.02	0.1
Weight-velocity (%)	-0.10	-0.18,-0.02	0.011	-6.16	-14.11,1.79	0.1
MOTOR DEVELOPMENT (months)						
Age at walking						
Walking	1.35	-0.073,2.78	0.06	1.30	-0.11,2.72	0.07
$Walking^2$	-0.057	-0.10,-0.01	0.02	-0.054	-0.10,-0.01	0.02
CHILDHOOD DEVELOPMENT (SD)						
Cognition	-0.32	-0.98,0.33	0.3	-0.41	-1.08,0.27	0.2
$Cognition^2$	-0.78	-1.25,-0.30	0.001	-0.77	-1.24,-0.30	0.001
Cognition*age (year)	0.07	0.021,0.11	0.004	0.067	0.02,0.11	0.003
$Cognition^2$ * age (year)	0.03	-0.01,0.06	0.09	0.024	-0.01,0.06	0.2
FATHER'S OCCUPATIONAL CLASS						
I & II	REF		0.08	REF		0.09
III	-0.62	-1.81,0.58		-0.77	-1.99,0.44	
IV and V	-1.55	-2.91,-0.18		-1.63	-3.08,-0.18	

+ P-values are given for the overall tests of associations

For peer review only - http://bmjopen.bmj.com/site/about/guidelines.xhtml

Supplementary Table 3. Estimates From Multilevel Models Showing Mean Differences in Grip Strength (kg) and Mean Differences in Grip Strength Change (kg/year) in 1,320 NSHD Women (3,069 observations) for Mutually Adjusted Childhood Factors. All Models Adjusted for Age Term and Standardised Adult Height.

	Adjusted for age term and height			Adjusted for age term and height and all childhood factors		
	Reg. Coeff.	95% CI	P-value+	Reg. Coeff.	95% CI	P-value+
BIRTHWEIGHT (kg)						
Birthweight	1.20	0.32,2.08	.007	1.05	0.24,1.86	0.01
Birthweight*age (year)	-0.07	-0.13,-0.002	.04	-0.07	-0.14,-0.01	0.03
GROWTH PARAMETERS						
Height -size (cm)	-0.14	-0.28,0.01	0.07	-0.15	-0.30,0.01	0.06
Height-tempo (%)	0.02	-0.041,0.08	0.6	0.02	-0.04,0.09	0.3
Height-velocity (%)	-0.01	-0.05,0.037	0.7	-0.01	-0.05,0.04	0.9
Weight-size (kg)	0.36	-0.02,0.75	0.06	0.25	-0.15,0.65	0.2
Weight-tempo (%)	0.00	-0.05,0.05	1.0	0.01	-0.05,0.06	0.8
Weight-velocity (%)	-0.05	-0.11,0.07	0.08	-0.03	-0.91,0.26	0.3
MOTOR DEVELOPMENT (months)						
Age at standing	-0.16	2.50,3.39	0.03	-0.14	-0.29,0.00	0.06
CHILDHOOD DEVELOPMENT (SD)						
Cognition	0.41	0.09,0.73	.01	0.42	0.07,0.78	0.02
FATHER'S OCCUPATIONAL CLASS						
I & II	REF		0.2	REF		0.2
III	-0.59	-1.65,0.47		-0.41	-1.38,0.57	
IV and V	0.22	-0.99,1.43		0.59	-0.55,1.72	
III *age (year)	-0.02	-0.10,0.06	0.2	-0.02	-0.10,0.05	0.1
IV and V*age (year)	-0.08	-0.17,0.01		-0.09	-0.18,0.004	

+ P-values are given for the overall tests of associations

For peer review only - http://bmjopen.bmj.com/site/about/guidelines.xhtml

Supplementary Table 4 Estimates From Multilevel Models Showing Mean Differences in Grip Strength (kg) and Mean Differences in Grip Strength Change (kg/year) by Each Adult Factor. All Models Adjusted for Age Term and Standardised Adult Height.

		Men					Women	
	n	Reg. Coeff.	95% CI	P-value	n	Reg. Coeff.	95% CI	P-value
a.BMI (per SD)[a]	1,528				1,524			
BMI		1.66	0.93,2.38	<0.001		-0.18	-0.42,0.049	0.1
BMI^2		-0.49	-0.80,-0.18	0.002		n/a		
BMI*age		-0.057	-0.11,-0.0056	0.03		n/a		
b. Health conditions at 53y	1,415				1,441			
Health conditions		-0.84	-1.70,0.19	0.05		-1.42	-1.89,-0.94	<0.001
Health conditions^2		-0.055	-0.12,0.0057	0.08		0.030	-0.0066,0.066	0.1
c. Smoker v. non-smoker [a]	1,516	-1.28	-2.38,-0.18	0.02	1,520	-0.17	-0.90,0.55	0.6
d. Leisure time physical activity[a]	1,521					1522		
Inactive		REF		0.003		REF		0.005
Intermediate		1.52	0.54,2.51			0.85	0.21,1.48	
Active		1.04	0.23,1.84			0.71	0.18,1.24	
e. Verbal memory[a]	1,489				1,508			
Verbal memory		-0.67	-1.24,-0.093	0.02		0.070	-0.31,0.45	0.7
Verbal memory*age		0.10	0.061,0.15	<0.001		0.032	0.0020,0.063	0.04
f. Qualifications (per step)	1,445	0.17	-0.13,0.48	0.3	1,444	0.38	0.16,0.59	0.001
g. Own social class at 53y[b]	1,511				1,518			
I and II		REF		0.001		REF		0.001
III		0.83	-0.50,2.15			-1.02	-1.63,-0.41	
IV & V		-3.20	-5.25,-1.15			-1.11	-1.89.-0.33	
III *age		-0.16	-0.25,-0.066	0.001		N/A		
IV & V * age		0.50	-0.097,0.20			N/A		

[a]included as time varying covariates; [b] illustrated in Web Figure 1.

P-values for sex interactions: BMI (intercept) P<0.001; BMI (quadratic) P=0.004; BMI (slope) P=0.01; health conditions (slope) P=0.05; smoker P=0.08; sex*age*occupational class P=0.001

For peer review only - http://bmjopen.bmj.com/site/about/guidelines.xhtml

Supplementary Table 5. Estimates From Multilevel Models Showing Mean Differences in Grip Strength (kg) by age at First Walking (in 1161men, 2515 observations) and Age at First Standing (in 1211women, 2709 observations) Adjusted for Age Term, Standardised Adult Height, and Then Additionally Adjusted for Each Set of Adult Factors in Turn

	Men: Age at walking (linear)			Men: Age at walking (quadratic)			Women: Age at standing		
	Reg. Coeff.	95% CI	P-value	Reg. Coeff	95% CI	P-value	Reg. Coeff	95% CI	P-value
Adjusted for age, adult height	0.96[b]	-0.62,2.54	0.2	-0.042[b]	-0.096,0.011	0.1	-0.16[b,c]	-0.31,-0.12	0.03
Additional adjustments									
BMI[a], BMI^2, BMI*age	1.02	-0.55,2.60	0.2	-0.044	-0.097,0.0095	0.1	-0.17	-0.32,-0.02	0.03
Health conditions 53y, Health conditions*age	0.99	-0.58,2.57	0.2	-0.044	-0.097,0.0094	0.1	-0.18	-0.33,-0.04	0.01
Educational qualifications	0.88	-0.70,2.46	0.3	-0.040	0.094,0.013	0.1	-0.18	-0.33,-0.03	0.03
Verbal memory[a], Verbal memory*age	0.96	-0.62,2.54	0.2	-0.042	-0.095,0.011	0.1	-0.16	-0.31,-0.01	0.03
Own social class 53y, Own social class*age	1.05	-0.53,2.63	0.2	-0.046	-0.099,0.0072	0.09	-0.16	-0.31,-0.02	0.03
Smoking and exercise[a]	0.90	-0.67,2.47	0.3	-0.041	-0.094,0.012	0.1	-0.17	-0.32,-0.23	0.02

[a]time varying covariates; [b] also adjusted for childhood cognition; [c] also adjusted for birthweight

For peer review only - http://bmjopen.bmj.com/site/about/guidelines.xhtml

STROBE 2007 (v4) Statement—Checklist of items that should be included in reports of *cohort studies*
Checklist completed for: Developmental and adult risk factors associated with decline in grip strength from midlife to old age: a British birth cohort study

Section/Topic	Item #	Recommendation	Reported on page #
**Title and abstract**	1	(*a*) Indicate the study's design with a commonly used term in the title or the abstract	Title & abstract
		(*b*) Provide in the abstract an informative and balanced summary of what was done and what was found	2
**Introduction**			
Background/rationale	2	Explain the scientific background and rationale for the investigation being reported	4-5
Objectives	3	State specific objectives, including any prespecified hypotheses	5
**Methods**			
Study design	4	Present key elements of study design early in the paper	5-7
Setting	5	Describe the setting, locations, and relevant dates, including periods of recruitment, exposure, follow-up, and data collection	5-7
Participants	6	(*a*) Give the eligibility criteria, and the sources and methods of selection of participants. Describe methods of follow-up	5-7
		(*b*) For matched studies, give matching criteria and number of exposed and unexposed	n/a
Variables	7	Clearly define all outcomes, exposures, predictors, potential confounders, and effect modifiers. Give diagnostic criteria, if applicable	6-7
Data sources/ measurement	8*	For each variable of interest, give sources of data and details of methods of assessment (measurement). Describe comparability of assessment methods if there is more than one group	*6-7*
Bias	9	Describe any efforts to address potential sources of bias	9
Study size	10	Explain how the study size was arrived at	5
Quantitative variables	11	Explain how quantitative variables were handled in the analyses. If applicable, describe which groupings were chosen and why	6-7, 8-9
Statistical methods	12	(*a*) Describe all statistical methods, including those used to control for confounding	8-9
		(*b*) Describe any methods used to examine subgroups and interactions	8-9
		(*c*) Explain how missing data were addressed	6-7,9
		(*d*) If applicable, explain how loss to follow-up was addressed	9
		(*e*) Describe any sensitivity analyses	9

For peer review only

**Results**			
Participants	13*	(a) Report numbers of individuals at each stage of study—eg numbers potentially eligible, examined for eligibility, confirmed eligible, included in the study, completing follow-up, and analysed	5-7, all tables
		(b) Give reasons for non-participation at each stage	5,16
		(c) Consider use of a flow diagram	-
Descriptive data	14*	(a) Give characteristics of study participants (eg demographic, clinical, social) and information on exposures and potential confounders	9, Table 1
		(b) Indicate number of participants with missing data for each variable of interest	Table 1
		(c) Summarise follow-up time (eg, average and total amount)	6
Outcome data	15*	Report numbers of outcome events or summary measures over time	6-12, all tables
Main results	16	(*a*) Give unadjusted estimates and, if applicable, confounder-adjusted estimates and their precision (eg, 95% confidence interval). Make clear which confounders were adjusted for and why they were included	9-12, all tables
		(*b*) Report category boundaries when continuous variables were categorized	All relevant tables
		(*c*) If relevant, consider translating estimates of relative risk into absolute risk for a meaningful time period	-
Other analyses	17	Report other analyses done—eg analyses of subgroups and interactions, and sensitivity analyses	12, all tables
**Discussion**			
Key results	18	Summarise key results with reference to study objectives	12-13
**Limitations**			
Interpretation	20	Give a cautious overall interpretation of results considering objectives, limitations, multiplicity of analyses, results from similar studies, and other relevant evidence	14-15
Generalisability	21	Discuss the generalisability (external validity) of the study results	15-16
**Other information**			
Funding	22	Give the source of funding and the role of the funders for the present study and, if applicable, for the original study on which the present article is based	24

*Give information separately for cases and controls in case-control studies and, if applicable, for exposed and unexposed groups in cohort and cross-sectional studies.

Note: An Explanation and Elaboration article discusses each checklist item and gives methodological background and published examples of transparent reporting. The STROBE checklist is best used in conjunction with this article (freely available on the Web sites of PLoS Medicine at http://www.plosmedicine.org/, Annals of Internal Medicine at http://www.annals.org/, and Epidemiology at http://www.epidem.com/). Information on the STROBE Initiative is available at www.strobe-statement.org.

For peer review only
